# The impact of team preferences on soccer offside judgments in laypersons: a quasi-experimental study

**DOI:** 10.1186/s41235-020-00253-2

**Published:** 2020-10-23

**Authors:** Peter Wühr, Frowin Fasold, Daniel Memmert

**Affiliations:** 1 Department of Psychology, TU Dortmund University, Emil-Figge-Str. 50, 44227 Dortmund, Germany; 2 Institute of Exercise Training and Sport Informatics, Department Cognitive and Team/Racket Sport Research, German Sport University Cologne, Cologne, Germany

**Keywords:** Football, Offside rule, Soccer, Team preference, Wishful seeing

## Abstract

The present study uses a quasi-experimental design to investigate the impact of team preferences on the accuracy of offside judgments. In Experiments 1 and 2, supporters of two German soccer clubs (i.e., Borussia Dortmund and FC Schalke 04) judged offsides in artificial scenes from a match between the clubs. We expected that supporters of both clubs would less frequently report the offside position of a forward from the preferred team. The results of Experiment 1 partly confirmed the predictions. Both groups reported the offside position of a yellow forward less frequently than that of a blue forward, and this effect was much larger for supporters of Borussia Dortmund than for supporters of Schalke 04. The difference between groups could be attributed to team preferences. The weaker effect of team preference in supporters of Schalke 04 was attributed to an unexpected perceptual effect that increased the accuracy of offside judgments for blue forwards in both groups. Experiments 2 and 3 showed the presumed effect of team preferences and the perceptual effect, respectively, in isolation. In summary, the results of our experiments provide evidence for (a) an effect of team preferences and (b) an effect of shirt–background contrast on offside judgments in soccer.

## Significance statement

A central component of the popular game soccer is the “offside” rule because it increases the difficulty of scoring goals and, thus, makes the game more interesting. According to that rule, a player P can only score a goal if—at the moment the ball is played to P—at least two opposing players are between P and the opponents’ goal. Judging offside is a difficult task, with offside decisions often being heavily disputed among the observers of a match. Everyday observation suggests that observers often tend to “see” offside situations in favor of their team. Our research investigated whether such “wishful seeing” can affect—or distort—the offside judgments of layperson observers. In two experiments, supporters of two German soccer clubs (Borussia Dortmund and Schalke 04) judged offside scenes from a virtual match between the two clubs. Most interestingly, we obtained evidence for an impact of team preferences on offside judgments, which reflected different response biases for forwards of the preferred versus the nonpreferred team. Moreover, and unanticipated, we observed an additional effect of shirt color on offside judgments, which most likely reflected differences in shirt–background contrasts. To conclude, whereas previous studies mostly focused on perceptual sources of errors in offside judgments, our study provides evidence for an impact of team preference on offside judgments. Future research could investigate whether similar (top-down) effects might occur in people with experience in soccer refereeing.

## Introduction

The offside rule is fundamental in modern soccer. According to the offside rule, a player P of the attacking team can only score a regular goal if—at the moment a teammate passes the ball to player P—player P is not closer to the opponents’ goal than the second-last defender, including the goalkeeper (cf. FIFA 2015, Laws of the Game, p. 36). The primary purpose of this rule is to make scoring goals more difficult for the players and, thus, more interesting for the observer. However, everyone familiar with soccer knows that judging offsides correctly is a difficult task. When a group of people is watching a soccer match, contradictory judgments are sometimes made of the same offside situation. Whereas some people saw a player in an offside position, others did not. Most interestingly, as many soccer fans may have already observed, different judgments of the same offside situations are often correlated with different team preferences. That is, supporters of the team that scored a goal often judge the position of the scoring player as not offside, whereas supporters of the opposing team judge the position of the same player as offside. How can this happen? Is it possible that supporters of opposing teams sometimes perceive the same game situation differently? Or do supporters simply tend to claim what benefits their team, independently of the facts?

In a now classic article, Hastorf and Cantril (1954) were the first to note striking differences between how members of Princeton University and Dartmouth College described a match between the football teams from both universities in 1951. The game was a rough one, with injuries and penalties on both sides, although Dartmouth was penalized more often than Princeton. Interestingly, when asked a week later, most Princeton students blamed Dartmouth for starting the “rough” play. In contrast, most Dartmouth students blamed both teams for starting the rough play. Similarly, after having watched a movie of the game, Princeton students reported twice as many infractions from the Dartmouth team than from their own team, whereas Dartmouth students reported slightly more offences from the Princeton team. Hence, the results of this report suggest that team preference may affect perceiving and/or recalling a sports event. In a later section, we will describe some follow-up research on the classic Hastorf and Cantril study.

The present study investigates the impact of team preferences on offside judgments in soccer. Therefore, we compared the offside judgments of two groups of participants, each supporting a famous German soccer team (i.e., Borussia Dortmund vs. F.C. Schalke 04), in displays depicting scenes from a match between these two clubs. Our general hypothesis was that strong team preferences might bias offside judgments in favor of the preferred team. That is, supporters of Borussia Dortmund might report the offside position of a player from Borussia Dortmund less often than of a player from Schalke 04, and supporters of Schalke 04 might show the opposite pattern. Moreover, we sought to determine if such differences in reporting offside positions of the preferred team compared to a non-preferred team might reflect a case of “wishful seeing” (cf. Bruner 1957; Dunning and Balcetis 2013) or, instead, a case of biased reporting.

### Psychological analysis of offside judgments

Judging offside in a soccer match is the task of a referee supported by two assistant referees, one on each side of the field of play. Each assistant referee (AR) is responsible for one half of the field of play; that is, if team A plays from left to right against team B, then AR 1 will be judging offside when team A attacks the goal of team B, whereas AR 2 will be judging offside when team B attacks the goal of team A. The AR signals a presumed offside position by lifting a flag; the final decision, however, remains with the chief referee.

From a psychological point of view, judging offside is a perceptual classification task where an infinite set of situations must be sorted into one of two categories: offside or not offside. In such a classification task, the observer can make two types of correct judgements and two types of errors (e.g., Macmillan and Creelman 2005). The correct judgments involve the classification of an actual offside situation as “offside” (called a “hit”) and the classification of an actual not-offside situation as “not offside” (called a “correct rejection”). The wrong judgments involve the classification of an actual offside position as “not offside” (called a “miss” or “non-flag error”) and the classification of an actual not-offside situation as “offside” (called “false alarm” or “flag error”).

According to *signal detection theory* (e.g., Green and Swets 1966; MacMillan and Creelman 2005), two major determinants of classification performance under uncertainty are sensitivity and response bias. With offside judgments, sensitivity refers to the ability of the observer to (perceptually) discriminate offside from non-offside situations. In contrast, the term “response bias” describes the preference for one or the other response, independently from sensitivity. For example, a supporter of Borussia Dortmund might prefer the “non-offside” response over the “offside” response when a forward of Borussia Dortmund is involved in a perceptually unclear offside situation. Signal detection theory provides formulas for computing the sensitivity index (*d*′) and the response-bias measure *c* from the results of a two-choice classification task (e.g., MacMillan and Creelman 2005).

### Previous research on offside judgments

Even highly skilled ARs in professional soccer games exhibit considerable error rates (varying, on average, between 10 and 20%) in their offside judgments, demonstrating that judging offsides is a difficult task (for a review, see Catteeuw et al. 2010a). Previous research suggests that professional ARs are more likely to conduct flag errors (false alarms) than non-flag errors (misses). This pattern was found in real games (e.g., Catteeuw et al. 2010a, b; Helsen et al. 2006; Oudejans et al. 2000, 2005) and in laboratory situations (e.g., Catteeuw et al. 2009; Gilis et al. 2008; Put et al. 2013). However, the preponderance of flag errors is not a universal observation. Some studies also found a preponderance of non-flag errors in both top-class assistant referees (e.g., Barte and Oudejans 2012; Catteeuw et al. 2010a; Mallo et al. 2012) and in layperson judgments of static pictures showing potential offside situations (Wühr et al. 2015).

Previous attempts to explain errors in offside judgments almost exclusively referred to perceptual limitations or perceptual biases in offside decisions. Belda Meruenda (2004) suggested that errors in offside judgments could result when ARs move their eyes from one player to another player. In particular, performing a saccade from the midfielder passing the ball to the forward receiving the ball takes time, during which the forward could have moved from an offside position into a not-offside position or vice versa. However, studies using eye-tracking methods (Catteeuw et al. 2009) or head-mounted camera systems (Oudejans et al. 2000) rejected this account.

Baldo et al. (2002) attributed errors in judging offside to a visual illusion called the *flash-lag effect*. In a typical task designed to show this effect, participants have to locate the position of a moving stimulus when a stationary event (the flash) occurs. The typical result is that participants misperceive the position of the moving stimulus in the direction of the movement (e.g., Müsseler et al. 2002). The task of judging offside is similar to the flash-lag task because ARs have to judge the position of a moving forward when a kick (a stationary event corresponding to the flash) occurs at a different position. The flash-lag hypothesis predicts a preponderance of flag errors (false alarms) because, when the forward moves towards the goal of the opposing team, ARs may misperceive the position of the forward in this direction. Instead, previous studies with static displays observed a preponderance of non-flag errors (e.g., Wühr et al. 2015), suggesting that flash-lag effects do not have a strong influence on offside judgments with static displays.

In their *optical error hypothesis*, Oudejans et al. (2005) describe how suboptimal viewing positions of the AR could also produce errors in offside judgments. ARs are encouraged to align with the offside line because this is considered the optimal position for judging offside (IFAB 2018, Practical Guidelines for Match Officials, p.184). However, studies have revealed that ARs do not perfectly align themselves with the offside line in a match but are often standing ahead or behind the offside line (e.g., Mallo et al. 2010; Oudejans et al. 2005). When the AR is standing ahead, or behind the offside line, the probability of flag errors (false alarms) or non-flag errors (misses) might increase depending on the particular spatial configuration of the AR, the second-last defender, and the forward (see Oudejans et al. 2005, for a detailed description).

To summarize, previous research has focused on perceptual sources of errors in offside judgments, whereas the possible impact of non-perceptual variables (e.g., attitudes, motives, needs, values) has been neglected. The present study provides a first step toward addressing non-perceptual variables by studying the possible impact of team preferences on soccer offside judgments.

### Wishful seeing

According to the dominant view in modern psychology, human perception is both selective and biased. Concerning selectivity, observers are widely agreed to consciously perceive only a small fraction of the information available to our sense organs, with (selective) attention playing a central role in selecting information for conscious perception and action (e.g., Allport 1987; Pashler 1998; Treisman 2009). Concerning bias, a growing body of empirical evidence suggests that conscious perception is the result of a compromise between bottom–up processing and top–down processing (e.g., Bar and Bubic 2014; Dunning and Balcetis 2013; Otten et al. 2017; Vetter and Newen 2014).

Following a study by Hastorf and Cantril (1954), several studies explored whether and how motivational or social variables affect the observation and recollection of a competitive sports event. Noting some methodological limitations, Boon and Davies (1996) conducted a conceptual replication of the Hastorf and Cantril study. Boon and Davis presented 25 videotaped excerpts from a soccer match between England and Scotland to a group of English students, who supported the English team, and a group of Scottish students, who supported the Scottish team. The excerpts showed incidents involving players from both teams, with an equal number of events showing offences of English and Scottish players but without showing the actual referee decisions. Participants were asked to make a referee decision on a seven-point scale, involving three decisions against an English player and three decisions against a Scottish player. Most importantly, the participants’ judgments were biased in favor of their preferred team.

Molenberghs et al. (2013) randomly assigned student participants to a “blue” or a “red” team. They observed that team membership biased judgments, with the speed with which members of both teams reached to press a button being affected. When participants performed the action–judgment task during functional magnetic resonance imaging (fMRI) measurement, neural responses in the inferior parietal lobule (IPL) in those participants who showed the bias in behavior were enhanced during the perception of own-group actions compared to actions of members of the competing group.

Recently, Huff et al. (2017) tested Hastorf and Cantril’s (1954) assertion that members and/or supporters of competing teams might literally “see” different versions of the same game. Therefore, Huff et al. investigated supporters of Borussia Dortmund and FC Bayern München while they watched the Champions League final between the two teams in 2013. In particular, the authors investigated participants’ perception (i.e., eye movements) during the match, their segmentation of the match into meaningful units, their memory for match events, and their emotional responses during and shortly after the match. Interestingly, results showed differences between the two fan groups in their emotional reactions to the match. Still, no differences were observed in eye movements, event segmentation, or memory for match events.

Whereas the studies described above investigated how partisanship (i.e., team membership or team preference) affects perception and memory of a competitive (sports) event, Balcetis and Dunning (2006, 2010; see Dunning and Balcetis 2013, for a review) investigated a different type of “wishful seeing,” namely, whether and how current desires affect the visual perception of objects. Balcetis and Dunning (2006) showed that participants’ desire (for a tasty beverage) affected their perception of ambiguous visual stimuli. In the critical trial of their experiments, participants were shown an ambiguous stimulus that could either be seen as the letter “B” or the number “13” on the computer screen. For half of the participants, letters were associated with a tasty beverage, and numbers were associated with an unpleasant beverage. In contrast, the opposite was true for the other half of participants. Most importantly, most of the first group reported having seen the letter B, whereas most of the second group reported having seen the number 13. Subsequent experiments replicated this finding and demonstrated that the participants’ mindset affected their perception of the ambiguous stimulus, not just the participants’ report. In a subsequent study, Balcetis and Dunning (2010) went on to show that participants’ desires can also affect distance judgments for desire-related objects. For example, in one of their experiments, the distance between the observer and a bottle of water was judged smaller by a group of thirsty participants than by a group of quenched participants. According to the authors, “perceiving desirable objects as closer can energize actions meant to obtain those objects” (p. 151).

In summary, previous studies on the effects of team membership or team preference on the perception and memory for a competitive (sports) event suggest that partisanship can bias judgments of rough play in favor of the preferred team (Boon and Davies 1996; Hastorf and Cantril 1954). Yet, no objective standard exists for evaluating rough play. Therefore, these observations most likely reflect different evaluations of an event that is perceived equally by supporters of competing teams. This interpretation is supported by Huff et al. (2017). They did not find differences in how supporters of two soccer teams watched, segmented, and remembered a significant match between the two teams. In contrast to these sports-related studies, research by Balcetis and Dunning (2006, 2010; Dunning and Balcetis 2013) suggests that people’s current desires (e.g., physiological needs) may bias the visual identification and localization of objects.

## The present study

The purpose of the present experiments was to test whether and how team preferences affect offside-judgments of laypersons. We compared the offside judgments of two groups of German participants, with one group supporting Borussia Dortmund and the other group supporting Schalke 04. We chose these two clubs because supporters of Borussia Dortmund are easily found at TU Dortmund University, and FC Schalke 04 is a traditional rival, which is located only 40 km away from Dortmund.

**Fig. 1:**
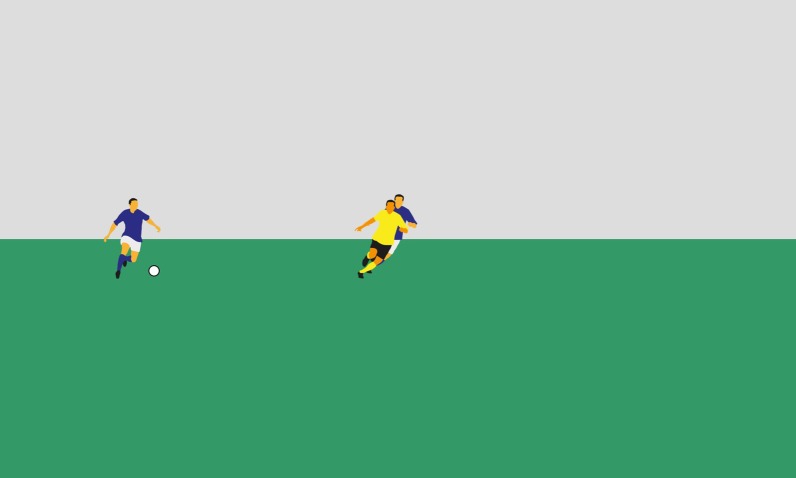
Example of a display used in Experiment 1. In this display, the blue team (resembling Schalke 04) attacks from left to right. The position of the yellow defender defines the offside line. Thus, the blue forward is in an offside position

The stimulus material consisted of static pictures showing scenes from a match between Borussia Dortmund and Schalke 04. Instead of using photographs, we constructed images with clipart figures to have maximal experimental control over the characteristics of the picture (see Fig. 1, for example). Using static pictures of an offside situation may simplify the situation compared to real games when a situation evolves, and observers might build up a mental model of the evolving situation. However, according to the “rules of the game,” only a single moment, that is, the moment when the ball is played to the forward, is crucial for the offside judgment, and our research focuses on how this very moment is judged. Hence, each participant was presented with pictures showing Borussia Dortmund attacking and pictures showing Schalke 04 attacking. Moreover, we varied the spatial distance between the forward of the attacking team and the defender, whose position defined the offside line. In every trial, the participants’ task was to decide whether the forward of the attacking team was in an offside position or not, and to report their judgments by pressing a key. The displays were shown for a brief duration to make the judgments difficult. The primary dependent variable was the percentage of correct judgments.

**Fig. 2:**
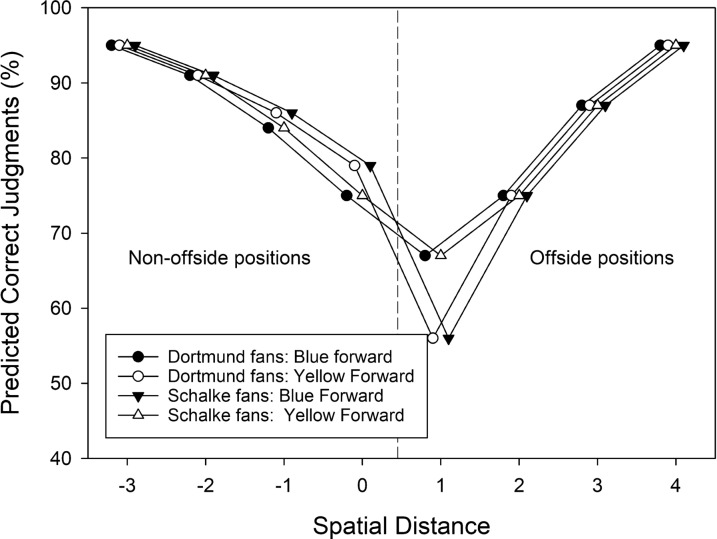
Predicted effects of observers’ team preferences (Dortmund vs. Schalke) on offside judgments for blue or yellow forwards. Note that spatial distances from − 3 to 0 refer to non-offside positions, whereas the spatial distances from 1 to 4 refer to offside positions

Our general hypothesis was that strong team preferences would bias offside judgments in favor of the preferred team. That is, supporters of Borussia Dortmund might less often report the actual offside position of a player from Borussia Dortmund than the actual offside position of a player from Schalke 04. Supporters of Schalke 04 should show the opposite pattern. Figure 2 shows hypothetical results illustrating our predictions. In Fig. 2, spatial positions from − 3 to 0 denote non-offside positions, whereas spatial positions from 1 to 4 denote offside positions. Figure 2 shows three predictions. First, hardly surprising, we predicted that the accuracy of correct offside judgments should be inversely related to the spatial separation between the forward and the defender (cf. Wühr et al. 2015). Hence, the accuracy of offside judgments should be lower at smaller distances. Second, consistent with our previous findings, we also predicted that in doubtful (i.e., difficult) situations, our layperson participants should have a preference for the “no-offside” response (cf. Fasold et al. 2017; Wühr et al. 2015). As a result, when the percentage of correct judgments is plotted as a function of spatial distance, the conditions with worst performance should not be symmetrically distributed around the smallest distance but should be shifted to the right of this condition. Third, different team preferences should lead to opposite patterns for blue versus yellow players, and these effects should be most visible in the most difficult conditions. In particular, supporters of Borussia Dortmund should report the offside positions of yellow forwards (unfilled circles in the right half of Fig. 2) less often than the offside positions of blue forwards (filled circles in the right half of Fig. 2). In addition, supporters of Borussia Dortmund might also report the non-offside positions of yellow forwards (unfilled circles in the left half of Fig. 2) more often than the non-offside positions of blue forwards (filled circles in the left half of Fig. 2). In contrast, supporters of Schalke 04 should report the offside positions of blue forwards (filled triangles in the right half of Fig. 2) less often than the offside positions of yellow forwards (unfilled triangles in the right half of Fig. 2). In addition, supporters of Schalke 04 might also report the non-offside positions of blue forwards (filled triangles in the left half of Fig. 2) more often than the non-offside positions of yellow forwards (unfilled triangles in the left half of Fig. 2).

In contrast to previous research on the impact of motivational or social variables on the observation of a competitive sports event, we used the methods of signal detection theory for localizing possible effects of partisanship (i.e., team preferences) on offside judgments. In particular, we determined whether team preferences can affect the ability to discriminate between offside and non-offside situations or change the preferences for offside versus non-offside responses. The application of signal detection analysis requires that the stimuli can be readily sorted in two (or more) categories based on a psychologically meaningful feature. This condition is met with offside situations but not so clearly with “rough play,” which has been the dependent variable in previous studies (e.g., Boon and Davies 1996; Hastorf and Cantril 1954). Because Huff et al. (2017) could not find any differences in how supporters of the opponent teams perceived and processed a soccer match, whereas earlier studies found biases in evaluating rough play (e.g., Boon and Davies 1996), we reasoned that team preferences, rather than sensitivity, are more likely to affect response bias.

## Experiment 1

Experiment 1 investigated the impact of team preferences on offside judgments in soccer situations. Two groups of participants were presented with pictures (cf. Fig. 1) showing situations from a soccer match between a team resembling Borussia Dortmund (yellow shirts, black shorts) and a team resembling Schalke 04 (blue shirts, white shorts). Participants in the first group were supporters of Borussia Dortmund, whereas participants in the second group were supporters of Schalke 04. The displays varied in two important ways. First, one half of the displays showed a situation where the yellow team attacked, whereas the other half of the displays showed a situation in which the blue team attacked. Second, in one half of the displays, the forward of the attacking team was not in an offside position, whereas, in the other half of the displays, the forward of the attacking team was in an offside position. The participants’ task was to decide as quickly as possible whether the forward in a display was in an offside position or not and to press a corresponding key. Notably, both the shirt color and the playing direction of the attacking team were blocked and, thus, predictable for the participants.

Our major hypotheses concerned the effects of team preferences on the accuracy of offside judgments. In particular, supporters of Borussia Dortmund should less often report the offside positions of yellow forwards compared to blue forwards. In contrast, supporters of Schalke 04 should less often report the offside positions of blue forwards compared to yellow forwards. This effect of team preferences on offside judgments should be most pronounced in the most difficult conditions where small spatial separations exist between the forward and the defender, and this effect might be accompanied by an opposite trend in judging non-offside positions.

### Methods

#### Participants

The participants were 84 students from different majors. Participants were recruited by posters addressing fans of Borussia Dortmund or Schalke 04. Fans of Borussia Dortmund were recruited at the campus of TU Dortmund University; fans of Schalke 04 were recruited at the campus of German Sports University Cologne. Thirty-six people (25 males, 11 females; mean age = 24.53 years, SD age = 3.89) responded to posters in Dortmund, and a questionnaire revealed that they all favored Borussia Dortmund (and disliked Schalke 04). Forty-eight people (46 males, 2 females; mean age = 27.31 years, SD age = 7.01) responded to posters in Cologne, but the questionnaire revealed that only 44 really favored Schalke 04 (and disapproved of Borussia Dortmund). All participants knew the offside rule, and six participants in the second sample (recruited in Cologne) had experience in refereeing football games. Each participant provided informed consent before the experiment. Further information on participants is reported in the results section. Participants received course credits if required.

#### Apparatus and stimuli

The experiment took place in a neutral laboratory room. Participants were seated in front of a 19-in. TFT color monitor without head fixation. The viewing distance was approximately 50 cm. From this viewing distance, 1 mm on the screen corresponds to approximately 0.12° of visual angle. A computer program, written with the software package *E-Prime 2.0* (Pittsburgh, PA: Psychology Software Tools), controlled the presentation of stimuli and registered keypress responses. The upward and downward arrow keys on a standard keyboard served as response keys; the two keys were aligned with the body midline.

The fixation point was a black plus sign in the center of the screen. The stimulus displays were 29.4 cm wide and 17.7 cm high. Each display showed a potential offside situation in which a midfielder from team A played the ball to a forward moving in the vicinity of a defender from team B. The players were colored clipart figures (Fig. 1). Each player was 30 mm high (3.44° of visual angle) and 21–24 mm wide (2.4°‬–2.8°); the ball had a diameter of 5 mm (0.57°). The players of one team wore the typical dress of Borussia Dortmund (i.e., yellow shirt [RGB values = 248, 234, 27; luminance ≈ 8.0 cd/m^2^] and socks, black shorts), whereas the players of the other team wore the typical dress of Schalke 04 (i.e., blue shirt [RGB = 43, 46, 131; luminance ≈ 0.8 cd/m^2^] and socks, white shorts). The upper half of the background was gray (RGB = 220, 220, 220; luminance ≈ 7.6 cd/m^2^); the lower half of the background was green (RGB = 50, 150, 100; luminance ≈ 2.8 cd/m^2^).

The defender was always presented at the screen center. The midfielder with the ball was presented in the left or right periphery at a distance of 13 cm (14.80°) from the defender. When the attacking team played from left to right, the midfielder appeared to the left; when the attacking team played from right to left, the midfielder appeared to the right. The forward was presented in the vicinity of the defender. Importantly, the position of the forward varied on both the vertical and the horizontal axis. Concerning vertical position, the forward was presented 2 mm above (and behind) the defender or 2 mm below (and in front of) the defender. The range of horizontal positions depended on the playing direction: when the attacking team played from left to right, the forward could appear 3, 2, 1, or 0 mm to the left or 1, 2, 3, or 4 mm to the right of the defender. In this case, the four positions to the right of the defender are offside. When, however, the attacking team played from right to left, the forward could appear 3, 2, 1, or 0 mm to the right or 1, 2, 3, or 4 mm to the left of the defender. In this case, the four positions to the left of the defender are offside.

The choice of dress colors may require some explanation. Neither the dress of Borussia Dortmund nor that of Schalke 04 has a typical yellow or blue color, respectively. Dress designs change from season to season, and dress colors often vary from season to season as well. For example, during the last 50 years, players of Borussia Dortmund wore shirts of varying yellows, but they also wore shirts of other colors (e.g., blue, red, black).^1^ Nevertheless, most people would agree that a yellow shirt and black trousers is the typical dress of Borussia Dortmund.

Similarly, most people would agree that a blue shirt and white trousers is the typical dress of Schalke 04. However, the “blue” color of the shirts has varied considerably in the past, and players of Schalke have worn other colors. Therefore, when starting our experiments, we chose yellow shirts and black trousers for the players of Borussia Dortmund and blue shirts and white trousers for players of Schalke 04, without worrying about the particular yellow or blue.

The combination of two playing directions (left → right; right → left) and two shirt colors of the attacking team (blue or yellow) produced four sets of stimulus displays. Each set contained 16 displays, from the factorial combination of two vertical positions (above/behind defender vs. below/in front of defender) and eight horizontal positions of the forward (− 3, − 2, − 1, 0, 1, 2, 3, 4).

We also constructed a short questionnaire containing 16 items. Items 1–5 asked for demographical details (i.e., age, gender, preferred hand, profession, and visual acuity). Items 6–8 asked whether participants have experience with playing soccer, with being a referee, or with being an assistant referee (yes/no). Item 9 asked about a participant’s interest in soccer on a scale from 0 (not at all) to 10 (extremely). Item 10 asked participants to indicate their favorite soccer team. Items 11–15 asked participants to rate their sympathy for each of five German soccer teams (F.C. Bayern München, Borussia Dortmund, VfL Wolfsburg, Schalke 04, SV Werder Bremen) on a scale from 0 (none) to 10 (extremely high). Item 16, finally, probed for an explicit bias in the offside judgment task (I favored the blue team; I favored neither team; I favored the yellow team).

#### Procedure

Participants were tested individually. After being seated, participants read and signed the informed consent sheet. When the research assistant had started the experiment, the instructions appeared on the screen. The instructions described the offside rule, presented two example displays (an offside situation and a no-offside situation), and explained the participant’s task. In particular, participants were asked to take the role of an ad-hoc AR in a match between Borussia Dortmund and Schalke 04. They were supposed to judge potential offside situations by pressing the upward key with the index finger of one hand for offside situations and the downward key with the index finger of the other hand for no-offside situations. The research assistant answered questions concerning the instructions and left the room after the practice block.

The experiment consisted of 10 blocks of 32 trials. Within each block, both the shirt color (blue vs. yellow) and the playing direction (left-to-right vs. right-to-left) of the attacking team were constant and, thus, predictable. Half of the participants were presented with displays on which the attacking team always played from left to right. In contrast, the other half of the participants were presented with displays on which the attacking team always played from right to left. However, for each participant, the dress color of the attacking team changed after five blocks. That is, each participant was presented with the displays from two sets (*see* above), with the same playing direction, in different blocks of the experiment. The order of shirt colors of the attacking team (blocks 1–5: blue; blocks 6–10: yellow or vice versa) was counterbalanced across participants. Block 1 and block 6 were practice blocks because they were the first blocks with a particular shirt color of the attacking team; the remaining blocks were test blocks.

Each experimental trial started with an empty (gray) screen for 1000 ms. Next, the fixation cross was shown for 500 ms at the screen center. To prevent eye movements of participants during the stimulus presentation, we chose a short presentation duration by presenting the stimulus display for 80 ms. The display was followed by another empty (gray) screen for 2000 ms (the duration times were derived from the research of Wühr et al. 2015). During the 2-s response interval, the computer registered all keypresses and their reaction times. No feedback was obtained concerning the accuracy or speed of responses. After the response registration period, the next trial started immediately. After the last block, participants filled out a short questionnaire, were debriefed, and thanked for participation. An experimental session lasted, on average, 30 min.

#### Design and data analysis

We planned three sets of analyses on the results of Experiment 1. The first analysis was an omnibus test (ANOVA) of how the three independent variables *group* (two levels), *attacking team* (two levels), and *spatial distance* (eight levels) affected the percentages of correct offside judgments. Attacking team and spatial distance were varied within participants. In this analysis, we were mainly interested in the presence of a three-way interaction, reflecting lower accuracy in judging offside (or higher accuracy in judging non-offside) for forwards from the preferred team, compared to forwards from the nonpreferred team, at a short distance. The second analysis was a three-factorial omnibus test (ANOVA) with group, attacking team, and spatial distance as independent variables, and RTs as the dependent variable. The primary purpose of this analysis was to check for speed–accuracy trade-offs, and therefore, the results are not discussed in much detail. Third, we analyzed the impact of group (two levels) and attacking team (two levels) on the sensitivity index *d*′ and the response-bias measure *c*. For computing these measures, we merged data from the four non-offside positions to one non-offside condition and from the four offside positions to one offside condition, respectively.

Two technical variables were independently counterbalanced across participants: (a) the playing direction of the attacking team and (b) the order of shirt colors of the attacking team. When describing the results of three-factorial ANOVAs involving the factor *spatial separation*, we report the Greenhouse–Geisser corrected *p* and the corrected degrees-of-freedom where appropriate. Regarding pairwise tests, we report the results of a Welch test^2^ (with corrected degrees of freedom) instead of Student’s test if the results of Levene’s test indicate unequal variances. Moreover, we report partial η^2^ as an effect size estimate for *F* tests and Hedges’ *g* as an effect size estimate for *t* tests (cf. Fritz et al. 2012; Lakens 2013).

### Results

#### Questionnaire data

All participants in the first group indicated Borussia Dortmund as their favorite team, whereas only 44 participants in the second group indicated Schalke 04 as their favorite team. The four remaining participants (i.e., 102, 112, 129, 131 in the dataset) from the second group were excluded from analysis. Moreover, we excluded six additional participants from the second group (i.e., 113, 122, 124, 139, 140 and 147 in the dataset) because they had experience in refereeing football matches. We had two reasons for doing this. First, no supporter of Borussia Dortmund in our sample had experience in refereeing football matches, and we wanted to match the two groups for this feature. Second, we suspected that experience in refereeing might reduce the impact of team preferences on offside judgments because referees are expected to be neutral on their job. Hence, dropping ten cases left 38 participants in the group of supporters of Schalke 04.

Participants in both groups indicated high interest in soccer (supporters of Borussia Dortmund: *M* = 8.56, SD = 1.21; supporters of Schalke 04: *M* = 7.34, SD = 1.96). When compared to supporters of Schalke 04, supporters of Borussia Dortmund revealed a higher preference for Borussia Dortmund, Welch’s *t*(44.4) = 47.366, *p* < .001, *g* = 10.770, and a higher preference for Werder Bremen, Welch’s *t*(71.0) = 4.686, *p* < .001, *g* = 1.092, but a lower preference for Schalke 04, Welch’s *t*(64.8) = − 32.646, *p* < .001, *g* = − 7.651, and for Bayern Munich, Welch’s *t*(68.9) = − 3.371, *p* = .001, *g* = − 0.788. No difference was observed in the preferences for VfL Wolfsburg, Welch’s *t*(71.4) = − 1.212, *p* = .229, *g* = − 0.282. Finally, a large majority of participants in both groups declared having favored neither team during the offside judgment task (supporters of Borussia Dortmund: 91.67%; supporters of Schalke 04: 94.74%). Descriptives of questionnaire results are also reported in Table 1.

**Table 1:** Descriptive statistics (mean, standard deviation, minimum, and maximum) of the preference data observed in Experiments  1 and 2

	Experiment 1		Experiment 2	
	Supporters of Borussia Dortmund	Supporters of Schalke 04	Supporters of Borussia Dortmund	Supporters of Schalke 04
Football	8.56 (1.21; 6, 10)	7.34 (1.96; 3, 10)	8.61 (1.20; 6, 10)	8.53 (1.41; 4, 10)
Bayern München	1.03 (1.50; 0, 7)	2.39 (1.97; 0, 7)	1.23 (1.61; 0, 6)	2.07 (2.07; 0, 8)
Borussia Dortmund	9.86 (0.35; 9, 10)	0.74 (1.13; 0, 7)	9.77 (0.43; 9, 10)	1.67 (2.15; 0, 7)
FC Schalke 04	1.22 (1.20; 0, 5)	9.29 (0.89; 7, 10)	1.58 (2.01; 0, 7)	9.70 (0.65; 8, 10)
VfL Wolfsburg	3.25 (1.95; 0, 7)	3.79 (1.88; 0, 10)	3.39 (1.80; 0, 7)	2.80 (1.67; 0, 7)
SV Werder Bremen	5.97 (1.77; 1, 9)	4.11 (1.66; 0, 7)	6.00 (2.19; 0, 8)	5.77 (2.01; 0, 8)

Participants rated their interest in football and their preferences for each of five German football clubs on an eleven-point scale (0–11)

#### Percentage of correct judgments (PC)

**Fig. 3:**
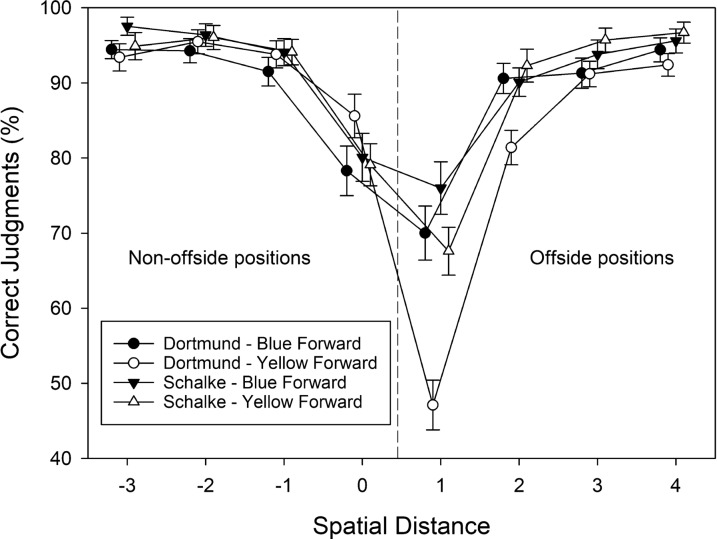
The percentage of correct offside judgments observed in Experiment 1, plotted as a function of spatial distance (between forward and defender), participants’ team preference (Dortmund vs. Schalke), and shirt color of the attacking team (blue vs. yellow). Error bars represent standard errors between participants. For each level of spatial distance, data points are jittered to increase readability

Trials without a response (*M* = 0.69, SD = 1.11, for blue forwards; *M* = 0.89, SD = 1.24, for yellow forwards) were removed from further analysis. The percentages of correct offside judgments were subjected to a three-factorial ANOVA with the between-subjects variable group, and the within-subjects variables attacking team and spatial distance. The corresponding cell means are shown in Fig. 3. All three main effects were significant. A just significant main effect of group indicated that supporters of Schalke 04 provided more accurate judgments (*M* = 90.05%, SD = 9.25) than supporters of Borussia Dortmund (*M* = 86.57%, SD = 12.17), *F*(1, 72) = 3.870, MSE = 56.475, *p* = .050, $\eta_{\rm partial}^{2}$ = 0.052. The main effect of attacking team reflected more accurate judgments when the blue team attacked (*M* = 89.33, SD = 8.53) than when the yellow team attacked (*M* = 87.29, SD = 12.96), *F*(1, 72) = 14.305, MSE = 85.560, *p* < .001, $\eta_{\rm partial}^{2}$ = 0.166. The main effect of spatial distance referred to the finding that the accuracy of offside judgments decreased when spatial distance decreased with a minimum at level 1, *F*(2.03, 145.98) = 87.309, MSE = 653.71, *p* < .001, $\eta_{\rm partial}^{2}$ = 0.548.

The most important finding was a significant three-way interaction, *F*(3.58, 257.77) = 6.626, MSE = 156.846, *p* = .012, $\eta_{\rm partial}^{2}$ = 0.084. To unravel the source of the three-way interaction, we first ran separate two-way ANOVAs to check whether the attacking team × spatial separation interaction was significant for both groups (cf. Fig. 3). In fact, the two-way interaction was significant for supporters of Borussia Dortmund, *F*(7, 245) = 18.586, MSE = 82.880, *p* < .001, $\eta_{\rm partial}^{2}$ = 0.347, and for supporters of Schalke 04, *F*(7, 259) = 2.831, MSE = 77.703, *p* = .007, $\eta_{\rm partial}^{2}$ = 071. Supporters of Borussia Dortmund provided more accurate (non-offside) judgments for yellow forwards compared to blue forwards at position 0, *t*(35) = − 2.269, *p* = .030, *g* = 0.378, but less accurate (offside) judgments for yellow forwards compared to blue forwards at position 1, *t*(35) = 6.459, *p* < .001, *g* = 1.077, and at position 2, *t*(35) = 4.498, *p* < .001, *g* = 0.750. We did not correct the pairwise comparisons for alpha-error inflation because we had actually predicted differences in the accuracy of offside judgments between blue vs. yellow forwards in both groups of participants at small distances. Therefore, the pairwise comparisons were considered planned comparisons. The remaining comparisons were not significant, all *t*s(35) < 1.6, all *p*s > 0.120, and all *g*s < 0.3. Supporters of Schalke 04 provided more accurate (offside) judgments for yellow forwards compared to blue forwards at position 1, *t*(37) = 2.587, *p* = .014, *g* = 0.420. The remaining comparisons were not significant, all *t*s(37) < 1.8, all *p*s > 0.090, and all *g*s < 0.3. Finally, we compared the differences in accuracy scores for blue versus yellow forwards at each spatial separation between the two groups. This set of comparisons revealed that supporters of Borussia Dortmund showed a stronger decrement in the accuracy of offside judgments for yellow forwards compared to blue forwards at positions 1 and 2 than did supporters of Schalke 04, both *t*s(72) > 3.00, both *p*s < 0.005, and both *g*s > 0.7. The remaining comparisons were not significant, all *t*s(72) < 2.00, all *p*s > 0.050, and all *g*s < 0.45.

Finally, two out of three possible two-way interactions were also significant. The group × spatial distance interaction, *F*(2.03, 145.98) = 3.963, MSE = 653.71, *p* = .021, $\eta_{\rm partial}^{2}$ = 0.052, indicated that supporters of Borussia Dortmund (*M* = 58.51%, SD = 19.91) were markedly less accurate than supporters of Schalke 04 (*M* = 71.79%, SD = 20.33) when the forward was slightly offside (i.e., at distance level 1), whereas performance was quite similar for the remaining distances (all pairwise differences < 5%). The attacking team × spatial distance interaction, *F*(3.58, 257.77) = 15.764, MSE = 156.846, *p* < .001, $\eta_{\rm partial}^{2}$ = 0.180, indicated that offside judgments were markedly less accurate when the yellow forward was at position 1 (*M* = 57.33%, SD = 22.39) than when the blue forward was at position 1 (*M* = 72.98%, SD = 21.45), whereas performance was quite similar for the remaining distances (all pairwise differences < 4%).

#### Signal detection analysis

The sensitivity index *d*′ describes the ability of discriminating offside from non-offside situations. We computed *d*′ for each combination of group (i.e., supporters of Borussia Dortmund vs. supporters of Schalke 04) and attacking team (blue vs. yellow) and subjected these measures to a two-factorial ANOVA with group as a between-subjects factor and attacking team as a within-subjects factor. The corresponding means are shown in Table 2. The main effect of group was marginally significant, reflecting higher sensitivity (*M* = 2.686, SD = 0.547) in supporters of Schalke 04 than in supporters of Borussia Dortmund (*M* = 2.403, SD = 0.673), *F*(1, 72) = 3.986, MSE = 0.744, *p* = .050, $\eta_{\rm partial}^{2}$ = 0.052. A significant main effect for attacking team indicated higher sensitivity for blue forwards (*M* = 2.643, SD = 0.703) than for yellow forwards (*M* = 2.447, SD = 0.648), *F*(1, 72) = 10.208, MSE = 0.138, *p* = .002, $\eta_{\rm partial}^{2}$ = 0.124. The two-way interaction was not significant, however, *F*(1, 72) = 0.864, MSE = 0.138, *p* = .356, $\eta_{\rm partial}^{2}$ = 0.012.

**Table 2:** Sensitivity index *d*′ as observed in Experiments 1 and 2 as a function of group (supporters of Borussia Dortmund vs. supporters of Schalke 04) and attacking team (blue vs. yellow)

	Supporters of Borussia Dortmund		Supporters of Schalke 04	
	Blue forward	Yellow forward	Blue forward	Yellow forward
Experiment 1	2.529 (0817)	2.277 (0.721)	2.756 (0.564)	2.617 (0.527)
Experiment 2	3.237 (0.847)	3.283 (0.946)	3.118 (0.542)	3.223 (1.199)

Standard deviations are given in parentheses

The measure *c* indicates the direction and size of a response bias. We subjected the bias measures to a two-factorial ANOVA with group as a between-subjects factor and attacking team as a within-subjects factor. The corresponding means are shown in Table 3. The main effect for group was not significant, *F*(1, 72) = 3.858, MSE = 0.147, *p* = .053, $\eta_{\rm partial}^{2}$ = 0.051. A significant main effect for attacking team indicated a stronger preference for the non-offside response when the yellow team attacked (*M* = 0.247, SD = 0.299) than when the blue team attacked (*M* = 0.105, SD = 0.332), *F*(1, 72) = 17.780, MSE = 0.042, *p* < .001, $\eta_{\rm partial}^{2}$ = 0.198. Importantly, the two-way interaction was also significant, *F*(1, 72) = 10.694, MSE = 0.042, *p* = .002, $\eta_{\rm partial}^{2}$ = 0.129. This interaction reflected the fact that only supporters of Borussia Dortmund were more reluctant to give offside responses with yellow forwards compared to blue forwards, *t*(35) = − 5.970, *p* < .001, *g* = − 0.995; supporters of Schalke 04 did not show this effect, *t*(37) = − 0.614, *p* = .543, *g* = − 0.099.

**Table 3:** Response bias index *c* as observed in Experiments 1 and 2 as a function of group (supporters of Borussia Dortmund vs. Supporters of Schalke 04) and attacking team (blue vs. yellow)

	Supporters of Borussia Dortmund		Supporters of Schalke 04	
	Blue forward	Yellow forward	Blue forward	Yellow forward
Experiment 1	0.112 (0.301)	0.363 (0.238)	0.098 (0.362)	0.129 (0.311)
Experiment 2	0.086 (0.292)	0.181 (0.271)	0.316 (0.185)	0.091 (0.200)

Standard deviations are given in parentheses

#### Reaction time (RT)

**Fig. 4:**
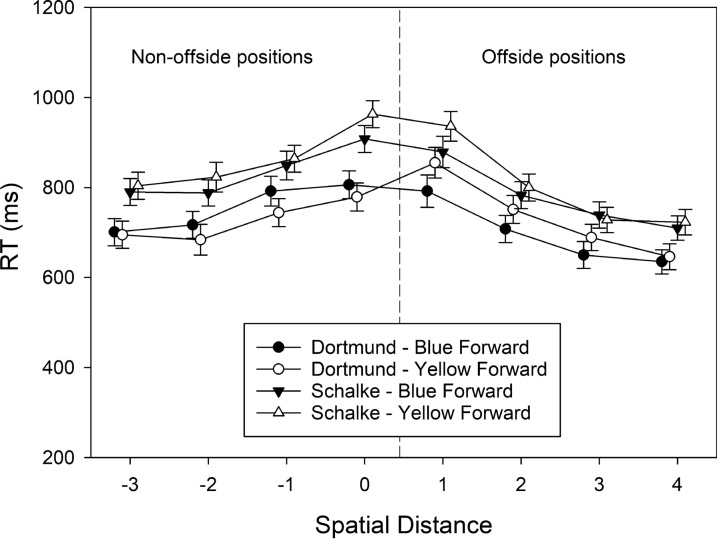
The RTs of correct offside judgments observed in Experiment 1, plotted as a function of spatial distance (between forward and defender), participants’ team preference (Dortmund vs. Schalke), and shirt color of the attacking team (blue vs. yellow). Error bars represent standard errors between participants. For each level of spatial distance, data points are jittered to increase readability

The RTs of correct judgments were also subjected to a three-factorial ANOVA with the between-subjects variable group, and the within-subjects variables attacking team and spatial distance. The corresponding cell means are shown in Fig. 4. The significant main effect of group indicated that supporters of Borussia Dortmund made faster judgments (*M* = 728 ms, SD = 177) than supporters of Schalke 04 (*M* = 818 ms, SD = 206), *F*(1, 72) = 6.703, MSE = 22,386.856, *p* = .012, $\eta_{\rm partial}^{2}$ = 0.085. The main effect of attacking team was not significant, *F*(1, 72) = 1.218, MSE = 51,950.283, *p* = .273, $\eta_{\rm partial}^{2}$ = 0.017. The main effect of spatial distance referred to the finding that RTs increased when spatial distance decreased, with a maximum at levels 0 and 1, *F*(3.58, 257.91) = 50.589, MSE = 27,508.081, *p* < .001, $\eta_{\rm partial}^{2}$ = 0.413.

The two-way interactions of group × attacking team, *F*(1, 72) = 0.531, MSE = 51,950.283, *p* = .469, $\eta_{\rm partial}^{2}$ = 0.007, and of group × spatial distance, *F*(3.58, 257.91) = 1.843, MSE = 27,508.081, *p* = .129, $\eta_{\rm partial}^{2}$ = 0.025, were not significant. The two-way interaction of attacking team × spatial distance, *F*(5.35, 384.96) = 2.892, MSE = 8,501.162, *p* = .012, $\eta_{\rm partial}^{2}$ = 0.039, and the three-way interaction, *F*(5.35, 384.96) = 3.264, MSE = 8501.162, *p* = .006, $\eta_{\rm partial}^{2}$ = 0.043, were significant, however. To unravel the source of the three-way interaction, we conducted separate two-way ANOVAs for each attacking team with group and spatial separation as independent variables. When the blue team attacked, supporters of Borussia Dortmund (*M* = 725 ms, SD = 66) generally responded faster than supporters of Schalke 04 (*M* = 806 ms, SD = 68), *F*(1, 72) = 4.738, MSE = 201,979.121, *p* = .033, $\eta_{\rm partial}^{2}$ = 0.062, and this difference in performance was independent of spatial distance, *F*(3.42, 246.12) = 0.323, MSE = 22,046.803, *p* = .834, $\eta_{\rm partial}^{2}$ = 0.004. When, however, the yellow team attacked, a main effect of group was observed, indicated by faster responses by supporters of Borussia Dortmund, *F*(1, 72) = 7.069, MSE = 208,160.865, *p* = .010, $\eta_{\rm partial}^{2}$ = 0.089, but a significant group × spatial separation interaction, *F*(4.28, 308.36) = 4.454, MSE = 16,023.435, *p* = .001, $\eta_{\rm partial}^{2}$ = 0.058, revealed that the difference varied with spatial position. In fact, supporters of Borussia Dortmund responded faster than supporters of Schalke 04 for non-offside positions (mean difference in RTs = 133 ms), *t*(72) = − 3.347, *p* = .001, but not for offside positions (mean difference in RTs = 67 ms), *t*(72) = − 1.757, *p* = .083.

### Discussion

Experiment 1 investigated the potential impact of team preferences on offside judgments in layperson participants. Supporters of Borussia Dortmund and supporters of Schalke 04 classified offside and non-offside situations in pictures from a virtual match between Borussia Dortmund and Schalke 04. We had predicted that different team preferences should produce a complementary pattern of errors in offside judgments. In particular, supporters of Borussia Dortmund should less often report the offside position of a yellow forward as opposed to a blue forward. In contrast, supporters of Schalke 04 should show the opposite pattern. Moreover, the effect of team preferences on offside judgments should be most pronounced in the difficult conditions where the spatial separation between forward and defender is small.

The supporters of Borussia Dortmund showed the predicted pattern of results. At small spatial separations (i.e., conditions 1 and 2), these participants reported offside positions less often for yellow forwards than for blue forwards. In addition, at the smallest separation of − 1, supporters of Borussia Dortmund also reported non-offside positions more often for yellow forwards than for blue forwards. A corresponding response bias was also found in the measure *c* for supporters of Borussia Dortmund. Notably, this pattern of findings cannot be attributed to different speed–accuracy trade-offs for blue versus yellow forwards because the independent variables had consistent effects on RTs and accuracy scores. That is, for a given spatial separation, both speed and accuracy indicated better performance for the same shirt color of the attacking team.

The supporters of Schalke 04 did not show the predicted pattern of findings. At the smallest spatial separation (i.e., 1), these participants also reported offside positions less often for yellow forwards than for blue forwards. Hence, in qualitative terms, the supporters of both Borussia Dortmund and Schalke 04 favored the yellow team over the blue team in their offside judgments. Most importantly, however, the results of Experiment 1 suggest an effect of different team preferences on offside judgments because supporters of Borussia Dortmund produced a much stronger advantage for the yellow team over the blue team than did the supporters of Schalke 04. Supporters of Schalke 04 did not show a significant response bias *c*. But why was the effect of team preference not strong enough in supporters of Schalke 04 to produce an advantage for the blue team, while the effect of team preference was strong enough in supporters of Borussia Dortmund to produce a (strong) advantage for the yellow team?

A possible first answer to the above question might be that supporters of Borussia Dortmund favor their team much more than supporters of Schalke 04 favor their team. This account, however, is unlikely for two reasons. First, supporters of both teams had a similarly high preference for their team and an equally low preference for the other team. Second, a smaller own-team preference in supporters of Schalke 04 could not explain why this group produced an, albeit small, advantage for the yellow team in their offside judgments.

A possible second answer to the above question could be that, in addition to team preferences, a second—unanticipated—variable had affected the offside judgments in Experiment 1 and overshadowed the impact of team preferences. Suppose that the second variable would always (i.e., regardless of team preference) increase the probability of reporting the offside position of a blue, as compared to a yellow, forward. In this case, team preference and the second variable would have worked in the same direction in supporters of Borussia Dortmund and produced a significant advantage for the yellow over the blue team (as observed). In contrast, in supporters of Schalke 04, team preference and the second variable would have worked in opposite directions and eliminated (or slightly reversed) the advantage for the blue over the yellow team (as observed).

We can only speculate about the nature of the variable that could have overshadowed the effects of team preferences on offside judgments in Experiment 1. A likely candidate could be a visual property of the displays that made it easier to correctly judge offside for blue compared to yellow forwards, which is reflected in higher sensitivity (*d*′) for the former task compared to the latter. In fact, the dress colors for the displays of Experiment 1 were chosen somewhat arbitrarily in an attempt to resemble the “typical” colors of Borussia Dortmund and Schalke 04. The background colors were taken from previous studies (i.e., Wühr et al. 2015). This choice of dress colors might have rendered blue forwards more salient than yellow forwards or yellow forwards less salient than blue forwards. In fact, when the contrasts are defined as Weber’s fraction, the stimulus background contrast for the blue shirt (i.e., the absolute value of − 0.895) was larger than for the yellow shirt (0.053). Similarly, when contrasts are defined as Michelson ratios, the contrast was larger for the blue shirt (0.810) than for the yellow shirt (0.026). In turn, this difference in salience might have increased the difference in the accuracy of offside judgments for blue versus yellow forwards in supporters of Borussia Dortmund and decreased (i.e., slightly reversed) the difference in the accuracy of offside judgments for blue versus yellow forwards in supporters of Schalke 04.

The two subsequent experiments were attempts to demonstrate in isolation the two effects that were presumably at work in Experiment 1. In Experiment 2, we aimed to demonstrate the effects of team preferences in isolation, attempting to prevent the perceptual effect by matching the brightness contrasts between the shirts and the background. In Experiment 3, we aimed at demonstrating the perceptual effects of different shirt–background contrasts in isolation.

## Experiment 2

Experiment 2 was an attempt to observe possible effects of team preferences on offside judgments without interference from the “perceptual” variable, which might have overshadowed the results in Experiment 1. Therefore, we replicated Experiment 1, but selected new colors for the shirts and the background to match the brightness contrasts between the shirts and the background (for details, see the Methods section). Besides using different colors, we made two additional changes to the experimental paradigm. First, whereas a constant display-presentation time (i.e., 80 ms) had been used in Experiment 1, we used variable display-presentation times in Experiment 2 and adapted the display presentation time to each participant’s performance. The primary purpose of this change was to reduce ceiling and floor effects in the accuracy of offside judgments. Second, we doubled the number of trials (from 320 to 640) and tested participants in two sessions, one for each color of the attacking team. Increasing the number of observations should produce more reliable observations and provide more space for adapting presentation time to performance. If matching the contrasts between shirts and background removes or eliminates the impact of perceptual effects on offside judgments, we should now observe the impact of team preferences in isolation.

### Methods

#### Participants

Participants were recruited by posters distributed over the campus of TU Dortmund University. Different posters explicitly addressed fans of Borussia Dortmund or Schalke 04. In total, 74 persons (mostly students) responded to our posters, and 67 persons completed both sessions. A power analysis with G*Power (Faul et al. 2007) revealed that a sample size of 32 participants (16 per group) would be required to allow detection of an interaction between a within-subjects factor (eight levels) and a between-subjects factor (two levels) with high power (*β* = 0.95, *α* = 0.05). For this analysis, we assumed an effect size of partial eta^2^ = 0.10, which was observed for the critical three-way interaction in Experiment 1. With this result in mind, we decided to collect at least 30 participants per group, similar to Experiment 1. Thirty-five participants (seven females, 28 males; mean age = 23.74 years, SD age = 6.61) declared to be supporters of Borussia Dortmund, and another 32 participants (five females, 27 males; mean age = 22.35 years, SD age = 2.60) declared to be supporters of Schalke 04. One quarter of the participants in each group consisted of active soccer players (at the amateur level). All participants knew the offside rule, but only a small fraction (i.e., two) of each group had experience in refereeing football games. Because the number of participants with experience in refereeing was equal (and small) in both groups, we did not exclude these participants as we did in Experiment 1. However, we excluded one participant from the second group (i.e., 104 in the dataset) because this participant revealed almost equal sympathy for Bayern Munich, Borussia Dortmund, and Schalke 04 in our questionnaire. Each participant provided informed consent before the experiment. Further information on the participants is reported in the results section. Participants received course credits or 5 Euro for completing the experiment.

#### Apparatus and stimuli

**Fig. 5:**
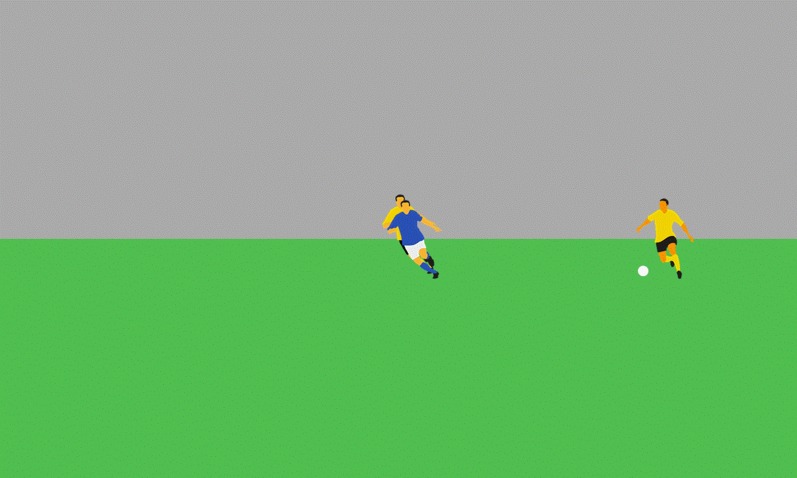
Example of a display used in Experiment 2. In this display, the yellow team (resembling Borussia Dortmund) attacks from right to left. The position of the blue defender defines the offside line. Thus, the yellow forward is in an offside position

We used the same apparatus as in Experiment 1. The structure of the displays was similar to Experiment 1, but the colors of figures and background were changed (cf. Figure 5). In particular, the blue shirt was made brighter (RGB = 40, 80, 180; approximately 1.5 cd/m^2^), whereas the yellow shirt was made darker (RGB = 240, 210, 0; approximately 6.5 cd/m^2^). Moreover, the upper gray background was made darker (RGB = 170, 170, 170; 4.0 approximately cd/m^2^), whereas the lower green background was made brighter (RGB = 80, 190, 80; approximately 4.0 cd/m^2^). When these measures were expressed as Weber’s fraction, we were able to match the absolute values of the shirt–background contrasts (*C*_W_ =|0.625|) in luminance. Moreover, when the shirt–background contrasts were defined as Michelson ratios, the shirt–background contrasts for the blue shirt (*C*_M_ = 0.455) and the yellow shirt (*C*_M_ = 0.238) were more similar than in Experiment 1. Note that the colors of the shirts were now taken from real shirts. In particular, the yellow color was taken from the BVB home shirt worn in season 2015/2016; the blue color was taken from the Schalke 04 home shirt worn in season 2014/2015.

#### Procedure

The experimental procedure in Experiment 2 was the same as in Experiment 1, with the following exceptions. First, compared to Experiment 1, the number of blocks (and trials) was doubled. Hence, Experiment 2 involved two sessions with 12 blocks of 32 trials in each session, and the shirt color of the attacking team varied between sessions. The two sessions took place on different days separated by, on average, 1 week. The first two blocks in each session were practice, followed by ten experimental blocks. Second, whereas a constant display duration had been used in Experiment 1, display duration was adapted to each participant’s performance in Experiment 2. We started with a duration of 100 ms in the first block. At the end of each block, the number of correct offside judgements was computed. If the percentage of correct judgments was between 60 and 90, the display duration was maintained. If the percentage of correct judgments was below 60%, the display duration was increased by the one screen-refresh rate (16.67 ms). If the percentage of correct judgments was above 90%, the display duration was decreased by the one screen-refresh rate. Participants were not informed about this feature of the experiment. Otherwise, the structure of blocks and trials was identical to Experiment 1.

For each participant, the direction of the attacking team (from left to right or from right to left) remained constant within blocks and across sessions, whereas the (shirt color of the) attacking team varied between sessions. Both the direction of the attacking team and the order of shirt colors across sessions were independently varied between participants.

#### Design and data analysis

The design and data analysis of Experiment 2 followed the same lines as for Experiment 1. After an initial screening, the data from six participants (four from group 1 [subjects 11, 19, 25, and 31 in the dataset] and two from group 2 [subjects 103 and 135 in the dataset]) were excluded from further analysis because their average percentage of correct judgments was close to guessing. That is, the overall percentage of correct response was between 48 and 51% for each of these participants; for the remaining participants, the percentage of correct responses was at least 60%. Excluding these participants, however, did not affect the overall pattern of results.

### Results

#### Questionnaire data

Participants in both groups indicated high interest in soccer (supporters of Borussia Dortmund: *M* = 8.61, SD = 1.20; supporters of Schalke 04: *M* = 8.53, SD = 1.41). Of course, all participants in the first group indicated Borussia Dortmund as their favorite team, whereas all participants in the second group indicated Schalke 04 as their favorite team. When compared to the supporters of Schalke 04, the supporters of Borussia Dortmund revealed a higher preference for Borussia Dortmund, Welch’s *t*(31.2) = 20.230, *p* < .001, *g* = 5.262, and a lower preference for Schalke 04, Welch’s *t*(36.4) = − 21.335, *p* < .001, *g* = − 5.391. No differences were observed in preferences for Bayern Munich, Welch’s *t*(54.7) = − 1.770, *p* = .082, *g* = − 0.455; Werder Bremen, Welch’s *t*(58.8) = 0.433, *p* = .666, *g* = 0.112; and VfL Wolfsburg, Welch’s *t*(58.9) = 1.321, *p* = .192, *g* = 0.338. Finally, a large majority of participants in both groups declared having favored neither team during the offside judgment task (supporters of Borussia Dortmund: 94%; supporters of Schalke 04: 88%). Descriptives of questionnaire results are also shown in Table 1.

#### Percentage of correct judgments (PC)

Trials without a response (*M* = 0.62, SD = 0.94, for blue forwards; *M* = 0.43, SD = 0.64, for yellow forwards) were removed from further analysis. For both groups of participants, the average display durations did not differ for blue and yellow forwards, Wilcoxon’s *W* = 134, *p* = .442, *g* = − 0.219 for supporters of Borussia Dortmund, and Wilcoxon’s *W* = 69.5, *p* = .499, *g* = − 0.235 for supporters of Schalke 04.

**Fig. 6:**
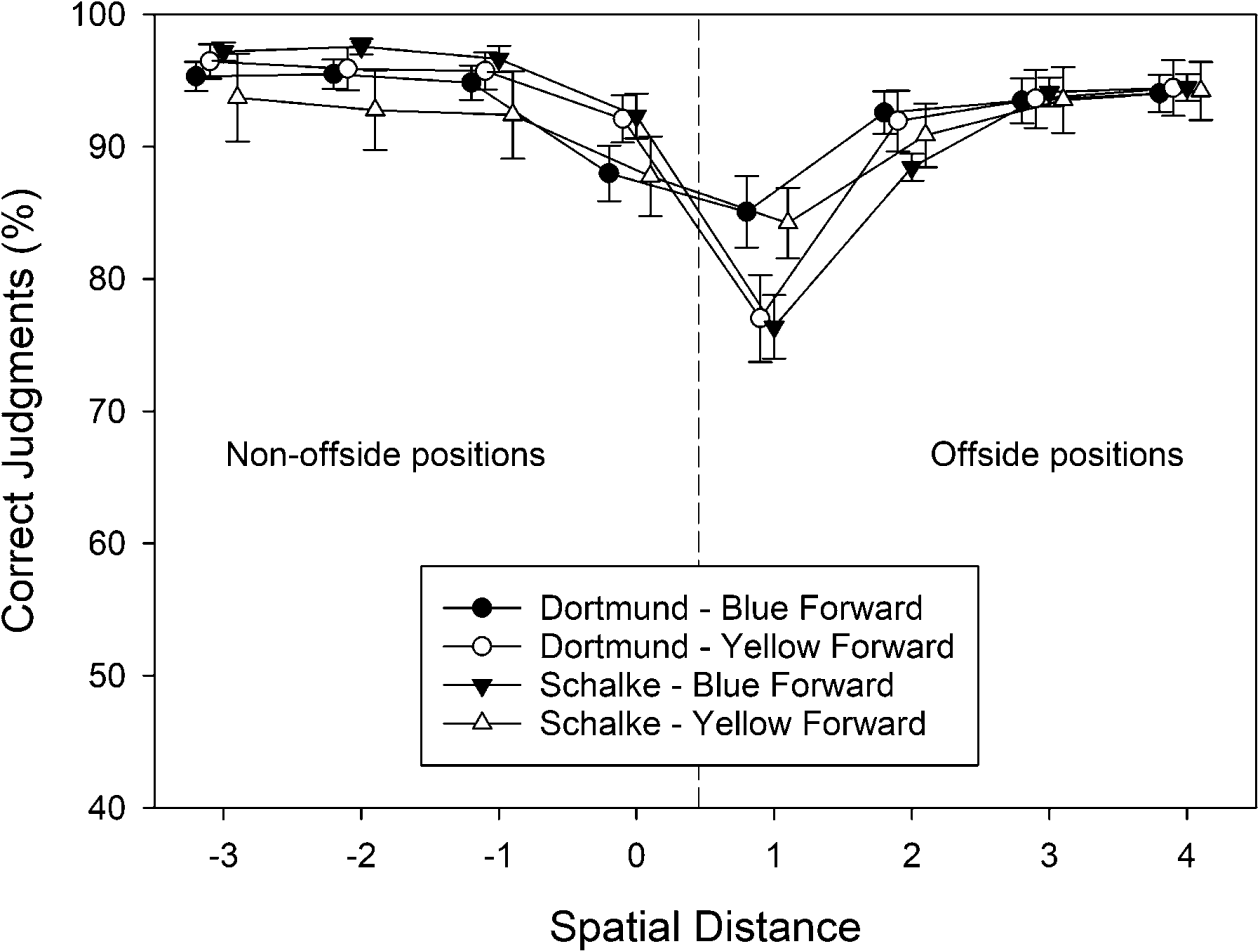
The percentage of correct offside judgments observed in Experiment 2, plotted as a function of spatial distance (between forward and defender), participants’ team preference (Dortmund vs. Schalke), and shirt color of the attacking team (blue vs. yellow). Error bars represent standard errors between participants. For each level of spatial distance, data points are jittered to increase readability

The percentages of correct offside judgments were subjected to a three-factorial ANOVA with the between-subjects variable group, and the within-subjects variables attacking team and spatial distance. The corresponding cell means are shown in Fig. 6. The significant main effect of spatial distance referred to the finding that the accuracy of offside judgments decreased when spatial distance decreased with a minimum at level 1, *F*(2.49, 144.51) = 61.472, MSE = 137.098, *p* < .001, $\eta_{\rm partial}^{2}$ = 0.515. The main effects of group, *F*(1, 58) = 0.082, MSE = 59.820, *p* = .776, $\eta_{\rm partial}^{2}$ = 0.001, and attacking team, *F*(1, 58) = 0.191, MSE = 438.433, *p* = .664, $\eta_{\rm partial}^{2}$ = 0.003, were not significant.

Again, the most important finding was the significant three-way interaction of attacking team × group × spatial distance, *F*(2.67, 154.82) = 12.199, MSE = 94.363, *p* < .001, $\eta_{\rm partial}^{2}$ = 0.174. None of the other interactions was significant, all *F* < 1.0, all *p* > .40. To unravel the source of the three-way interaction, we first ran separate two-way ANOVAs to check whether the attacking team × spatial separation interaction was significant for both groups (cf. Fig. 6). In fact, the two-way interaction was significant for supporters of Borussia Dortmund, *F*(2.55, 76.62) = 4.670, MSE = 109.75, *p* = .007, $\eta_{\rm partial}^{2}$ = 0.135, and for supporters of Schalke 04, *F*(2.66, 74.42) = 8.870, MSE = 83.316, *p* < .001, $\eta_{\rm partial}^{2}$ = 241. Supporters of Borussia Dortmund provided somewhat more accurate (non-offside) judgments for yellow forwards compared to blue forwards at position 0, *t*(30) = − 1.845, *p* = .075, *g* = − 0.377, and *less* accurate (offside) judgments for yellow forwards compared to blue forwards at position 1, *t*(30) = 2.504, *p* = .018, *g* = 0.488. Again, we did not correct the pairwise comparisons for alpha error inflation because we had actually predicted differences in the accuracy of offside judgments between blue vs. yellow forwards in both groups of participants at small distances. Therefore, the pairwise comparisons were considered planned comparisons. The remaining comparisons were not significant, all *t*s(30) < 1.2, all *p*s > 0.25, and all *g*s < 0.2. Supporters of Schalke 04 showed a different pattern. In fact, supporters of Schalke 04 provided numerically more accurate (non-offside) judgments for blue compared to yellow forwards, all *t*s(28) < 1.70, all *p*s > 0.10, and all *g*s < 0.50, at positions − 3 to 0, and *less* accurate (offside) judgments for blue forwards compared to yellow forwards at position 1, *t*(28) = − 2.663, *p* = .013, *g* = − 0.571. The remaining comparisons were not significant, all *t*s(28) < 1.2, all *p*s > 0.25, and all *g*s < 0.30.

#### Signal detection analysis

We computed the sensitivity index *d*′ for each combination of group (i.e., supporters of Borussia Dortmund vs. supporters of Schalke 04) and attacking team (blue vs. yellow) and subjected these measures to a two-factorial ANOVA with group as a between-subjects factor and attacking team as a within-subjects factor. The corresponding means are shown in Table 2. All *F* tests produced nonsignificant results, all *F*s(1, 58) < 1.

Next, we subjected the bias measure *c* to a two-factorial ANOVA with group as a between-subjects factor and attacking team as a within-subjects factor. The corresponding means are shown in Table 3. Neither main effect was significant, both *F*s(1, 58) < 2.60 and both *p*s > 0.10. Most importantly, however, the two-way interaction was significant, *F*(1, 58) = 15.768, MSE = 0.049, *p* < .001, $\eta_{\rm partial}^{2}$ = 0.214. This interaction reflected the fact that supporters of Schalke 04 were more reluctant to give offside responses to blue forwards compared to yellow forwards, *t*(28) = 4.655, *p* < .001, *g* = 0.958 (one-tailed), whereas supporters of Borussia Dortmund showed a trend in the opposite direction, *t*(30) = − 1.503, *p* = .072, *g* = − 0.313 (one-tailed).

#### Reaction time (RT)

**Fig. 7:**
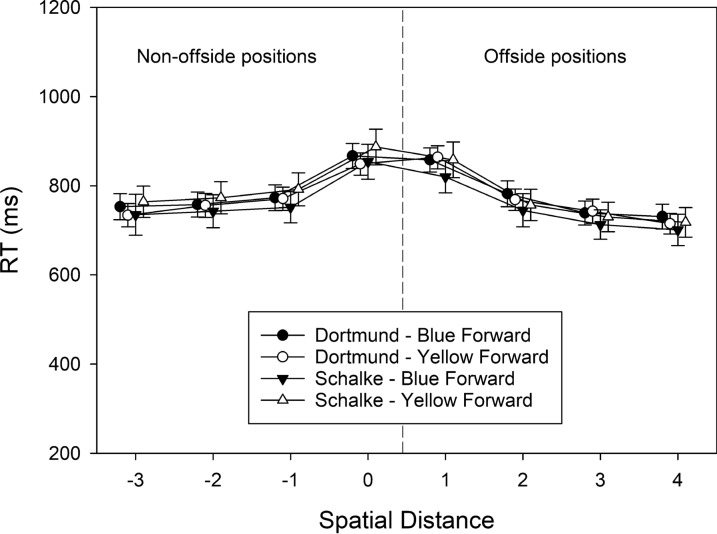
The RTs of correct offside judgments observed in Experiment 2, plotted as a function of spatial distance (between forward and defender), participants’ team preference (Dortmund vs. Schalke), and shirt color of the attacking team (blue vs. yellow). Error bars represent standard errors between participants. For each level of spatial distance, data points are jittered to increase readability

The RTs of correct judgments were also subjected to a three-factorial ANOVA with the between-subjects variable group and the within-subjects variables attacking team and spatial distance. The corresponding cell means are shown in Fig. 7. A main effect of spatial distance referred to the finding that RTs increased when spatial distance decreased from offside position − 3 to 0, remained high at position 1, and decreased again when spatial distance increased from offside position 1–4, *F*(2.79, 161.64) = 75.927, MSE = 11,434.526, *p* < .001, $\eta_{\rm partial}^{2}$ = 0.567. The main effects of group, *F*(1, 58) = 0.037, MSE = 22,244.087, *p* = .848, $\eta_{\rm partial}^{2}$ = 0.001, and attacking team, *F*(1, 58) = 0.350, MSE = 65,626.034, *p* = .556, $\eta_{\rm partial}^{2}$ = 0.006, were not significant. The interactions were also not significant: group × attacking team, *F*(1, 58) = 1.089, MSE = 65,626.034, *p* = .301, $\eta_{\rm partial}^{2}$ = 0.018; group × spatial distance, *F*(2.79, 161.636) = 1.294, MSE = 11,434.526, *p* = .279, $\eta_{\rm partial}^{2}$ = 0.022; attacking team × spatial distance, *F*(3.44, 199.30) = 0.785, MSE = 5349.668, *p* = .519, $\eta_{\rm partial}^{2}$ = 0.013; and group × attacking team × spatial distance, *F*(3,44, 199.30) = 0.450, MSE = 5,349.668, *p* = .743, $\eta_{\rm partial}^{2}$ = 0.008.

### Discussion

Experiment 2 attempted to isolate the effects of team preferences on offside judgements. In particular, by matching the brightness contrasts between shirt colors and the background, we tried to prevent an impact of this perceptual variable, which might have overshadowed the effects of team preferences on offside judgments in Experiment 1. Four results of Experiment 1 are noteworthy. First, in contrast to Experiment 1, the main effect of shirt color (i.e., attacking team) on several dependent measures (i.e., accuracy, d-prime, RT) was gone, suggesting that matching contrasts effectively reduced the presumed visual effect from Experiment 1. Second, both the accuracy data and the results of the signal-detection analysis (i.e., measure *c*) revealed a bias of reporting the preferred team less often in an offside position than the not preferred team in both groups of supporters. In particular, when compared to the not preferred team, the accuracy of offside judgments for the preferred team was decreased for offside judgments and increased for non-offside judgments in both groups of participants. Third, no effects of group (i.e., preferred team) on RTs were observed, excluding an explanation of the accuracy results in terms of different speed–accuracy trade-offs. Finally, after the experiment, most participants in both groups declared that they had favored neither team in the offside judgment task, suggesting that participants did not deliberately favor their preferred team.

## Experiment 3

Experiment 3 attempted to isolate the effect of the “perceptual” variable on offside judgments when using the displays from Experiment 1 by removing the possible impact of team preferences. Therefore, as in Experiment 1, but in contrast to Experiment 2, the contrast in brightness between blue shirts and the background was larger than the contrast in brightness between yellow shirts and the background. If the asymmetric contrasts in brightness produce an advantage in judging offside for blue forwards (and/or a disadvantage in judging offside for yellow forwards), this perceptual effect should dominate performance if the additional effects of team preferences were prevented. In Experiment 3, we prevented a systematic effect of team preferences on offside judgments by selecting only participants that favored neither Borussia Dortmund nor Schalke 04. Participants of Experiment 3 were allowed to favor all possible soccer clubs except for Borussia Dortmund and Schalke 04.

### Methods

#### Participants

The participants were 24 students of different majors (22 females, 2 males; mean age = 25.46 years, SD age = 4.88). Participants were recruited in lectures on the campus of the German Sports University in Cologne. However, only volunteers who were not fans of Borussia Dortmund or Schalke 04 were invited to participate in the experiment. The participants were highly interested in soccer (*M* = 8.17, SD = 1.67). Their favorite teams included 1 FC Cologne (5), FSV Mainz 05 (3), FC Bayern Munich (2), Real Madrid (2), Hamburger SV (2), Arsenal London (2), and eight other clubs. Approximately one half (i.e., 54%) of the participants were active soccer players (at the amateur level). All participants knew the offside rule, but only a small fraction (12%) had experience in refereeing soccer games. Each participant provided informed consent before the experiment.

#### Apparatus and stimuli

For Experiment 3, we used the same stimuli and an apparatus similar to the one used for Experiment 1.

#### Procedure

The experimental procedure in Experiment 3 was the same as in Experiment 1. In each block, both the shirt color (blue vs. yellow) and the playing direction (left-to-right vs. right-to-left) of the attacking team were constant and, thus, predictable. However, after five blocks, the shirt color of the attacking team (but not the playing direction) changed for another five blocks.

#### Design and data analysis

Experiment 3 had a 2 (attacking team) × 8 (spatial distance) within-subjects design. Two technical variables were independently counterbalanced across participants: (a) the playing direction of the attacking team and (b) the order of shirt colors of the attacking team. Again, we planned omnibus tests (ANOVAs) of the impact of the two independent variables on the percentage of correct offside judgments and RTs. In addition, we planned to analyze the impact of the attacking team (i.e., shirt color) on sensitivity (*d*′) and response bias (*c*).

Data from two participants (i.e., 7 and 8 in the corresponding dataset) were excluded from further analysis. For one participant, the average percentage of correct judgments was below guessing rate (i.e., 40%), whereas average performance was at least 70% for the remaining participants. The other participant had ceased the experiment before its end. Excluding these participants, however, did not affect the overall pattern of results.

### Results

#### Percentage of correct judgments (PC)

**Fig. 8:**
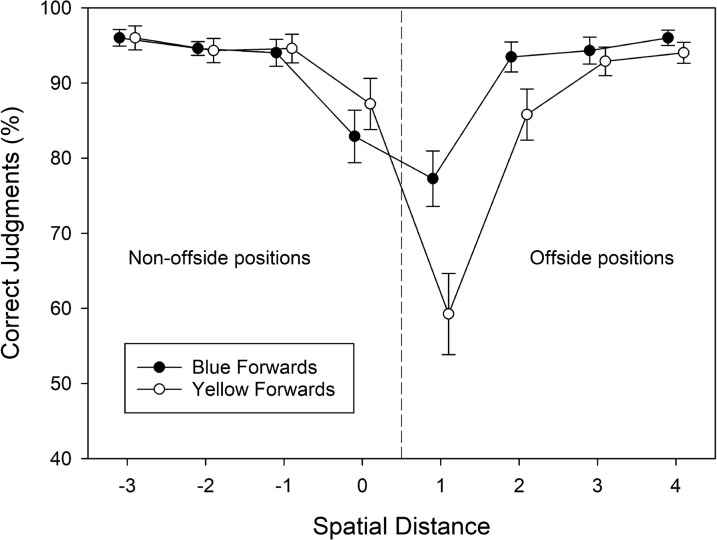
The percentage of correct offside judgments observed in Experiment 3, plotted as a function of spatial distance (between forward and defender) and shirt color of the attacking team (blue vs. yellow). Error bars represent standard errors between participants. For each level of spatial distance, data points are jittered to increase readability

Trials without a response (*M* = 1.43, SD = 1.43, for blue forwards; *M* = 1.65, SD = 2.13, for yellow forwards) were removed from further analysis. The percentages of correct offside judgments were subjected to a two-factorial ANOVA with attacking team and spatial distance as within-subjects variables. The corresponding cell means are shown in Fig. 8. The significant main effect of attacking team reflected more accurate judgments when the blue team attacked (*M* = 91.087, SD = 9.188) than when the yellow team attacked (*M* = 87.891, SD = 11.983), *F*(1, 21) = 8.298, MSE = 108.331, *p* = .009, $\eta_{\rm partial}^{2}$ = 0.283. The significant main effect of spatial distance referred to the finding that the accuracy of offside judgments decreased when spatial distance decreased with a minimum at level 1, *F*(2.36, 49.53) = 25.991, MSE = 452.314, *p* < .001, $\eta_{\rm partial}^{2}$ = 0.553. A significant two-way interaction of attacking team × spatial distance, *F*(3.24, 68.07) = 9.307, MSE = 132.713, *p* < .001, $\eta_{\rm partial}^{2}$ = 0.307, indicated that offside judgments were less accurate for yellow as compared to blue forwards both at position 1 (difference = 19%), *t*(21) = 4.664, *p* < .001, *g* = 0.894, and at position 2 (difference = 8%), *t*(21) = 2.694, *p* = .014, *g* = 0.600. The accuracy of offside judgments did not differ for other levels of spatial separation, all *t*s(21) < 1.70, all *p*s > 0.100, and all *g*s < 0.360.

#### Signal detection analysis

Participants were numerically better in discriminating offside positions for blue forwards (*M* = 2.903, SD = 0.678) than for yellow forwards (*M* = 2.688, SD = 0.722), *t*(22) = 1.913, *p* = .070, *g* = 0.307 (two-tailed). In addition, participants had a stronger preference for the non-offside response for yellow forwards (*M* = 0.308, SD = 0.278) than for blue forwards (*M* = 0.066, SD = 0.228), *t*(22) = − 4.499, *p* < .001, *g* = − 0.864 (two-tailed).

#### Reaction time (RT)

The RTs of correct judgments were also subjected to a two-factorial ANOVA with attacking team and spatial distance as within-subjects variables. The main effect of attacking team just missed conventional significance, *F*(1, 21) = 4.102, MSE = 48,962.656, *p* = .056, $\eta_{\rm partial}^{2}$ = 0.163. A significant main effect of spatial distance referred to the finding that RTs increased when spatial distance decreased in both directions with a maximum RT at distance condition 1, *F*(3.41, 71.56) = 18.786, MSE = 18,601.471, *p* < .001, $\eta_{\rm partial}^{2}$ = 0.472. The latter main effect was further qualified by a significant attacking team × spatial distance interaction, *F*(7, 147) = 5.803, MSE = 9081.597, *p* < .001, $\eta_{\rm partial}^{2}$ = 0.217. The two-way interaction demonstrated that only offside decisions (i.e., when the forward appeared at positions 1 to 4) were faster for blue forwards than for yellow forwards (mean difference = 86 ms), whereas RTs of non-offside decisions (i.e., when the forward appeared at positions − 3 to 0) were similar for both team colors (mean difference = 10 ms).

### Discussion

The results of Experiment 3 provide evidence for our hypothesis that a perceptual variable can affect offside judgments independently from team preferences when participants judge offside in the displays from Experiment 1. In Experiment 3, supporters of many different soccer clubs judged offside situations in displays showing situations from a (virtual) soccer match between two teams resembling Borussia Dortmund and Schalke 04. Consistent with our predictions, the results showed a decrease in the accuracy of offside judgments for yellow compared to blue forwards and a corresponding increase in latencies. This finding can be attributed to a perceptual variable, such as the different saliency of blue compared to yellow figures in our displays. We can exclude any consistent effect of different team preferences on performance in this experiment.

## General discussion

The primary aim of our experiments was to investigate the impact of different team preferences on offside judgments in soccer. To achieve this goal, supporters of Borussia Dortmund and supporters of Schalke 04 took the role of assistant referees and judged whether displays depicted an offside or a non-offside situation. The displays showed virtual scenes from a soccer match between players wearing yellow shirts and black shorts, resembling the colors of Borussia Dortmund, and players wearing blue shirts and white shorts, resembling the colors of Schalke 04. Our main prediction was that supporters of both teams should report an offside position of a forward from the preferred team less often than the offside position of a forward from the not preferred team. This effect should mainly show up in perceptually difficult situations (i.e., small distance conditions) and might be accompanied by a reverse effect in the accuracy of non-offside judgments.

The results of Experiment 1 partly confirmed our predictions. Consistent with our predictions, supporters of Borussia Dortmund reported the offside position of yellow forwards less often than those of blue forwards. Inconsistent with our predictions, however, was the finding that supporters of Schalke 04 showed a similar pattern. Importantly, however, when compared to Schalke 04, the supporters of Borussia Dortmund showed a much stronger decrease in the accuracy of offside judgments for yellow compared to blue forwards, suggesting an impact of team preferences on offside judgments in Experiment 1. To explain why team preferences were not strong enough to produce the expected pattern in supporters of Schalke 04, we suggested that a second effect might have—unexpectedly—overshadowed the impact of team preferences. In particular, we reasoned that if the second, probably visual, effect always increased the accuracy of offside judgments for blue compared to yellow forwards, this effect would add to the effect of team preferences in supporters of Borussia Dortmund, but counteract the effect of team preferences in supporters of Schalke 04. In particular, we speculated that the higher brightness contrast between yellow shirts and background, compared to the brightness contrast between blue shirts and background, facilitated offside judgments for players of Borussia Dortmund compared to players of Schalke 04.

Experiment 2 was an attempt to isolate the effect of team preferences on offside judgments. Here, supporters of Borussia Dortmund and Schalke 04 judged offside for similar displays as in Experiment 1, but for this experiment, the colors of the shirts and the background were chosen to match the contrasts between yellow and blue shirts and the background, respectively. We hypothesized that, under these conditions, the effects of team preferences should be stronger than possible effects of brightness contrast. The results showed the expected impact of team preferences on offside judgments: supporters of both teams now reported the offside position of forwards from their favored team less often than the offside position of forwards from the not preferred team.

Finally, Experiment 3 was an attempt to isolate the presumed effect of different brightness contrasts on offside judgments. Here, we simply removed the impact of specific team preferences on offside judgments by having supporters of many different soccer teams (except for Borussia Dortmund and Schalke 04) judge offsides with the displays from Experiment 1. As in Experiment 1, the results of Experiment 3 showed higher accuracy for judging offside positions of blue compared to yellow forwards, most likely reflecting the effect of a visual variable.^3^

### The impact of team preferences on offside judgments

Experiment 2 showed the expected effects of team preferences on offside judgments. Supporters of Borussia Dortmund and Schalke 04 reported an offside position of a player from their favorite team less often than they reported the offside position of a forward from the not preferred team. At the same time, supporters of both teams also reported the non-offside position of a forward from their favorite team more often than they reported the non-offside position of a forward from the not preferred team.

Most importantly, the effects of team preferences (i.e., partisanship) on the accuracy of offside judgments were not accompanied by reverse effects in RTs, excluding different speed-accuracy tradeoffs for the favorite and the non-preferred team as an account of the accuracy data. In addition, most participants (i.e., more than 85% of participants in both groups) declared after the experiment that they did *not* favor a team in their offside judgments, in contrast to the accuracy data. This finding might reflect a tendency for socially desirable answers, but it might also suggest that participants did not *deliberately* favor their team in their judgments. This hypothesis could be pursued in future research by, for example, testing the impact of error feedback on the pattern of judgments.

To investigate the possible locus of the effects of team preferences on offside judgments, we separately analyzed the effects of team preferences on the sensitivity measure *d*′ and the bias measure *c* (cf. MacMillan & Creelman, 2005). The sensitivity measure *d*′ describes the ability to discriminate offside from non-offside situations in our experiment. Effects on *d*′ may arise on any stage of processing devoted to perceiving and classifying the stimulus displays. In contrast, the bias measure *c* describes the preference for one or the other response, independently of *d*′. Hence, effects on *c* arise on the stage of selecting a response to the output of the stimulus classification process. Importantly, the results of Experiment 2 showed an effect of team preferences on *c* but not on *d*′, suggesting that team preference produced a response bias but did not affect perceiving and classifying the stimulus displays. Hence, these results indicate that when participants are watching a match of their favorite team, they are assigning different weights to the offside and the non-offside response depending on whether their favorite team or a nonpreferred team is attacking. In our experiment, the attacking team varied between blocks (or sessions in Experiment 2). Hence, conceivably, when participants had learned whether their favored team or the opposite team would attack in the current block of trials, they would change the weights of the response options in a way that benefits their team, producing the observed differences in response bias. These response biases have most likely been learned in the past because offside responses to forwards from the opposing team prevent undesired events (i.e., goals for the opponent). In contrast, non-offside responses to forwards from the favored team are sometimes followed by desired events (i.e., goals for the favored team).

Our experiments’ results complement previous research on the impact of team preference on the perception and memory for a competitive (sports) event. Some early studies have shown that partisanship could bias judgments of rough play in favor of the preferred team (Boon and Davies 1996; Hastorf and Cantril 1954). Because Huff et al. (2017) could not find differences in how supporters of two German soccer teams watched, segmented, and remembered an important match between the two teams, the effects of partisanship on evaluations of rough play, as shown in earlier studies, likely reflect different response biases too. In contrast to these sports-related studies, however, research by Balcetis and Dunning (2006, 2010; Dunning and Balcetis 2013) suggests that people’s physiological needs (e.g., thirst) may, in fact, bias the visual identification and localization of objects.

Similar to previous studies (e.g., Boon and Davies 1996; Huff et al. 2017), our results were obtained with a quasi-experimental design (cf. Reichardt 2019; Shadish et al. 2002). These quasi-experimental designs differ from experimental designs in that participants were not randomly assigned to the experimental conditions. Rather, participants assigned themselves to conditions (i.e., groups with different team preferences). Therefore, in principle, pre-experimental differences between the two groups besides team preferences could have affected the results. Regarding our results, however, thinking of a variable–other than team preference–that might have biased the offside judgments of the two groups in opposite directions is difficult. Nevertheless, the quasi-experimental character of our study should be kept in mind when interpreting the results.

### The impact of visual factors on offside judgments

Although not a primary aim of our research, novel evidence provided by our experiments suggests that differences in the brightness contrasts between player dresses and the background can also affect the accuracy of offside judgments. In particular, Experiment 1 produced partly unexpected results in that supporters of both teams showed less accurate offside judgments for yellow compared to blue forwards. Because supporters of Borussia Dortmund showed a much bigger disadvantage for the yellow forwards than supporters of Schalke 04, we assumed that two independent effects had produced the results of Experiment 1. The (expected) effect of team preference was assumed to decrease the accuracy of offside judgments for the preferred team, whereas an (unexpected) perceptual effect was assumed to increase the accuracy of offside judgments for blue forwards. Hence, for supporters of Borussia Dortmund, both effects would work in the same direction, producing a big decrement in accuracy of offside judgments for yellow as compared to blue forwards. In contrast, for supporters of Schalke 04, the two effects would work in opposite directions, cancelling out each other.

When analyzing the displays of Experiment 1, we noticed that—unwittingly—the yellow shirt and the gray background were more similar in brightness than the blue shirt and the background. Hence, we assumed that a larger brightness contrast between blue shirts and the background compared to yellow shirts, made the blue shirts more salient and facilitated offside judgments for blue forwards compared to yellow forwards. We tested this hypothesis in Experiments 2 and 3. In Experiment 2, we tried to eliminate the perceptual effect by matching the brightness contrasts between the two different shirts and the background. The results showed similar decrements in the accuracy of offside judgments for the preferred team in both groups of participants, suggesting that we successfully eliminated the perceptual effect from Experiment 1 and isolated the effects of team preferences on offside judgments in Experiment 2. Finally, in Experiment 3, we apparently isolated the perceptual effect when supporters of many different teams judged offside in the same displays as in Experiment 1. In sum, the results of Experiments 1–3 suggest that participants may better perceive the (offside vs. non-offside) position of a forward when the contrast in brightness between the forwards’ shirt and the background is larger than when this contrast is smaller.

Interestingly, signal detection analysis revealed higher sensitivity measures *d*′ for blue compared to yellow forwards in Experiments 1 and 3, in which the shirt–background contrast was higher for blue compared to yellow shirts. In contrast, no such effect was observed in Experiment 2, in which the shirt–background contrast was (almost) equal for blue as compared to yellow forwards. This pattern also suggests that the effect of shirt–background contrast arises at perceptual stages of processing. Unexpectedly, however, Experiment 3 also revealed a stronger preference (i.e., response bias) for the non-offside response with yellow as compared to blue forwards. At present, we do not have an explanation for this finding.

The literature contains only a single study that has addressed the impact of shirt colors on offside judgments in soccer. In this study, Krenn (2018) used videos of 1,530 matches from the first two divisions of the German Soccer Association to investigate the relationship between the dress colors of the defending and the attacking team, respectively, and the number of offside decisions. Krenn found that the frequency of offside decisions against the attacking team was reduced when the forwards wore black shirts. In addition, this author found that the frequency of offside decisions against the attacking team was increased when the defending team wore black or green shirts. Krenn assumed his findings were related to the reduced visibility of black or green shirts, against the background of a lawn or stadium. This interpretation is speculative, however, because Krenn had no information about the actual backgrounds in the game scenes that had been analyzed. Moreover, Krenn provides no information about the impact of dress colors on the accuracy of offside decisions but analyzed only the frequency of these decisions. At least, Krenn’s study provides evidence suggesting that color or brightness differences between shirts and the background may affect offside decisions. Our findings indicate that increasing the contrast between the background and the shirt color of the attacking player can improve the accuracy of offside judgments.

## Directions for future research

The results of the present experiments open up several avenues for future research. First, while we demonstrated that team preferences could affect offside judgments, subsequent research might explore the relationship between the strength of team preferences and the size of the response bias in offside judgments. For this purpose, using a regression-analytical approach, rather than a factorial design, and allowing for more variance in team preferences, as we had in our experiments, might be more useful.

Second, future research might also further investigate the effects of shirt–background contrasts on the accuracy of offside judgments. We consider this an important issue because knowing which dress-background combinations facilitate or impede offside judgments should be most interesting to players, referees, and football officials. When doing this, separate investigations into the impact of brightness contrasts and dress color effects might be useful. The pure impact of brightness contrasts could be investigated by using different gray levels instead of colors for players’ dresses and/or the background. The pure effects of colors might be investigated than by using equiluminant colors for players’ dresses and/or the background.

Finally, investigating whether and how different kinds of expertise (e.g., frequency of watching soccer, experience as AR or as a referee) might modulate the impact of team preferences or shirt colors on offside judgments might be interesting. For example, knowing whether experience as an AR decreases or eliminates the effect of team preferences on offside judgments would be interesting.

## Conclusions

The results of the experiments reported here go beyond previous research in two ways. First, Experiments 1 and 2 revealed the first evidence for an effect of participants’ team preferences on offside judgments: participants reported the offside position of a player from their preferred team less often (i.e., accurately) than the offside position of a player from the non-preferred team. Signal detection analysis revealed that team preferences produced a response bias, rather than affecting the ability to discriminate offside from non-offside situations. Second, the experiments also revealed evidence for an impact of the brightness contrast between the players’ shirts and the background on offside judgments: participants reported the offside position of a player more accurately when the contrast between the player’s shirt and the background was high rather than low. The present results provide interesting starting points and guidelines for subsequent research, in which both effects are further explored.

## Figures and Tables

**Fig. 9:**
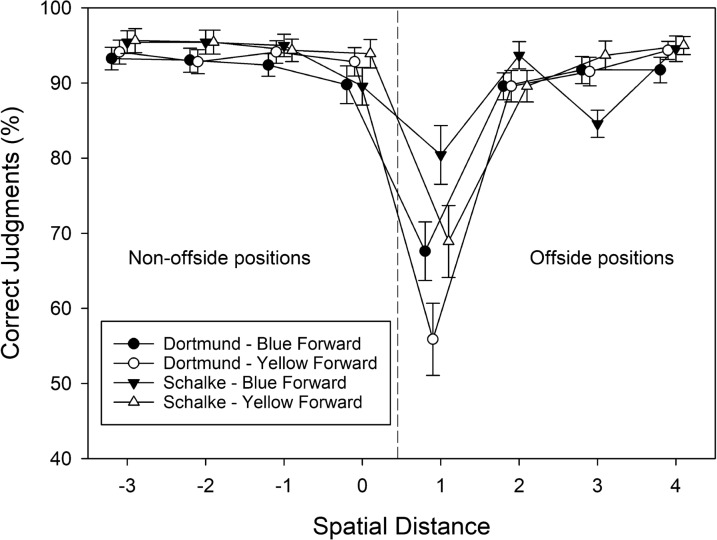
The percentage of correct offside judgments observed in Experiment 4, plotted as a function of spatial distance (between forward and defender), participants’ team preference (Dortmund vs. Schalke), and shirt colour of the attacking team (blue vs. yellow). Error bars represent standard errors between participants

**Fig. 10:**
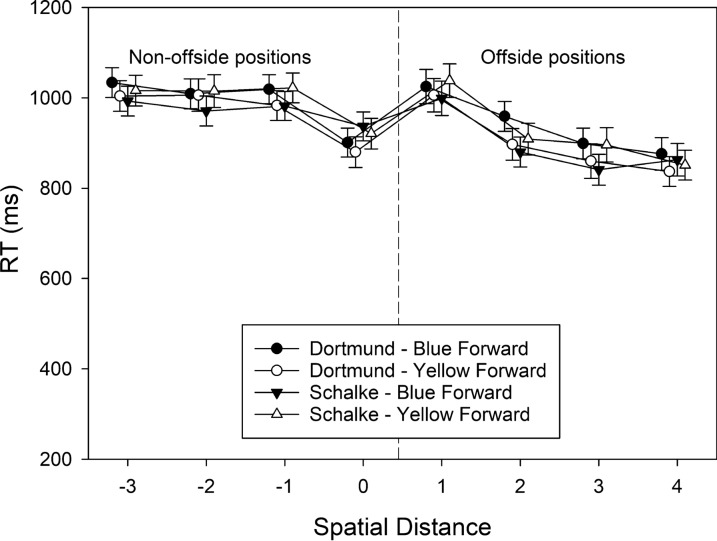
The RTs of correct offside judgments observed in Experiment 4, plotted as a function of spatial distance (between forward and defender), participants’ team preference (Dortmund vs. Schalke), and shirt colour of the attacking team (blue vs. yellow). Error bars represent standard errors between participants

## Data Availability

The raw data for Experiments 1, 2, and 3 are available in the “SowiDataNet|datorium” repository (https://dx.doi.org//10.7802/2046). The raw data for Experiment 4 and the questionnaire data are available from the first author upon request.

## Appendix

In this appendix, we report another experiment (called Experiment 4) that was not included in the final manuscript. In Experiment 4, participants (i.e. supporters of Borussia Dortmund and supporters of Schalke 04) judged offside in the same displays used in Experiment 1. The main difference between the two experiments was that the attacking team, which had been predictable in Experiment 1, was no longer predictable in this experiment. Accordingly, in Experiment 4, we mixed displays on which the blue team attacked in one direction and displays on which the yellow team attacked in the other direction within the same block.

We reasoned that removing the predictability of the attacking team in the next display would more strongly affect the impact of a motivational variable (i.e. team preference) on offside judgments than it should affect the impact of a perceptual variable (e.g., the different saliency of blue versus yellow forwards) on offside judgments. In particular, we assumed that the impact of a perceptual variable on offside judgments should depend *less* on the predictability of the attacking team than the impact of a motivational variable (team preference) on offside judgments. Therefore, we predicted that only the perceptual variable should affect the offside judgments of both groups of participants in Experiment 4. Hence, we should observe a similarly reduced accuracy of offside judgments (at small offside positions 1 and 2) for yellow as compared to blue forwards in both groups of supporters.

### Method

#### Participants

The participants were 50 students from different majors. Participants were recruited by (separate) posters addressing fans of Borussia Dortmund or Schalke 04 at the university campus. Twenty-five participants (10 female, 15 male; mean age = 25.32 years, SD age = 5.78) described themselves as being supporters of Borussia Dortmund, and another 25 participants (16 female, 9 male; mean age = 22.56 years, SD age = 3.37) described themselves as being supporters of Schalke 04. About one third (i.e. 35%) of the participants in each group were active soccer players (at the amateur level). All participants knew the offside rule, but only a small fraction of each group (12% versus 10%) had experience in refereeing football games. Each participant gave her/his informed consent before the experiment. Further information on the participants is reported in the results section.

#### Apparatus and stimuli

For Experiment 4, we used the same stimuli and a similar apparatus as for Experiment 1.

#### Procedure

The experimental procedure in Experiment 4 was the same as in Experiment 1, with the following exceptions. In contrast to Experiment 1, the shirt colour and the playing direction of the attacking team varied within blocks and, therefore, were no longer predictable. Rather, in Experiment 4, displays showing the yellow team attacking to one direction and displays showing the blue team attacking to the opposite direction were randomly mixed. Hence, in Experiment 4, the 32 different displays from two display sets were shown once in random order within each block. The experiment consisted of 11 blocks of 32 trials. The first block was practice, followed by ten test blocks. For half of the participants within both groups, the blue team played from left to right, and the yellow team played from right to left throughout the complete experiment. The directions were reversed for the other half of the participants within both groups.

#### Design and data analysis

Experiment 4 had the same 2 (*Group*) × 2 (*Attacking Team*) × 8 (*Spatial Distance*) mixed factorial design as Experiment 1, and the statistical analysis followed the same lines as for Experiment 1. The playing direction of the two teams (blue from left to right versus yellow from right-to-left or vice versa) was counterbalanced across participants.

After an initial screening, data from four participants (two from each group) were excluded from further analysis because their average percentage of correct judgments was close to guessing (i.e., the overall percentage of correct response was below 55% for each of the four participants; for the remaining participants, the percentage of correct responses was at least 65%). Excluding these participants, however, did not affect the overall pattern of results.

### Results

#### Questionnaire data

Participants in both groups indicated high interest in soccer (Supporters of Borussia Dortmund: *M* = 8.36, SD = 1.78; supporters of Schalke 04: *M* = 8.36, SD = 1.82). Of course, all participants in the first group indicated Borussia Dortmund as their favourite team, whereas all participants in the second group indicated Schalke 04 as their favourite team. When compared to the supporters of Schalke 04, the supporters of Borussia Dortmund revealed a higher preference for Borussia Dortmund, *t*(24.571) = 15.042, *p* < .001, *g* = 5.457, and a lower preference for Schalke 04, *t*(48) = − 25.647, *p* < .001, *g* = − 7.463. There were no differences in preferences for Bayern Munich, *t*(48) = − 0.349, *p* = .729, *g* = − 0.101, Werder Bremen, *t*(48) = − 0.001, *p* = .999, *g* = 0.001, or VfL Wolfsburg, *t*(48) = 0.095, *p* = .925, *g* = 0.028. Finally, a large majority of participants in both groups declared having favoured neither team during the offside-judgment task (Supporters of Borussia Dortmund: 92.0%; supporters of Schalke 04: 96.0%).

#### Percentage of correct judgments (PC)

The percentages of correct offside judgments were subjected to a three-factorial ANOVA with the between-subjects variable *Group*, and the within-subjects variables *Attacking Team* and *Spatial Distance*. The corresponding cell means are shown in Fig. 9. The significant main effect of *Spatial Distance* referred to the finding that the accuracy of offside judgments decreased when spatial distance decreased with a minimum at level 1, *F*(1.86, 81.93) = 53.726, MSE = 511.164, *p* < .001, $\eta_{\rm partial}^{2}$ = 0.550. In particular, the percentage of correct judgments significantly decreased from level 0 to the minimum at level 1, *F*(1, 44) = 45.980, *p* < .001, $\eta_{\rm partial}^{2}$ = 511, and then significantly increased with each step from level 1 to level 3, both *F*(1, 72) > 7.0, all *p* < .01, both $\eta_{\rm partial}^{2}$ > 0.140. The main effects of *Group*, *F*(1, 44) = 3.254, MSE = 35.728, *p* = .078, $\eta_{\rm partial}^{2}$ = 0.069, and *Attacking Team*, *F*(1, 44) = 3.183, MSE = 58.436, *p* = .081, $\eta_{\rm partial}^{2}$ = 0.067, were not significant.

A significant two-way interaction of *Attacking Team* × *Spatial Distance*, *F*(3.58, 257.77) = 15.764, MSE = 156.846, *p* < .001, $\eta_{\rm partial}^{2}$ = 0.180, indicated that offside judgments were less accurate when the yellow forward was at position 1 (*M* = 62.39%, SD = 23.47) than when the blue forward was at position 1 (*M* = 74.02%, SD = 19.74), whereas performance was quite similar for the remaining distances (all pairwise differences < 4%). The remaining interactions were not significant: *Group* × *Attacking Team*, *F*(1, 44) = 0.839, MSE = 58.436, *p* = .365, $\eta_{\rm partial}^{2}$ = 0.019, *Group* × *Spatial Distance*, *F*(1.86, 81.93) = 2.702, MSE = 511.164, *p* = .077, $\eta_{\rm partial}^{2}$ = 0.058, and *Attacking Team* × *Group* × *Spatial Distance*, *F*(4.98, 219.03) = 0.444, MSE = 56.194, *p* = .816, $\eta_{\rm partial}^{2}$ = 0.010.

#### Reaction time (RT)

The RTs of correct judgments were also subjected to a three-factorial ANOVA with the between-subjects variable *Group*, and the within-subjects variables *Attacking Team* and *Spatial Distance*. The corresponding cell means are shown in Fig. 10. The main effect of *Spatial Distance* referred to the finding that RTs increased when spatial distance decreased across the four offside positions, but remained relatively high across the four non-offside positions, *F*(4.03, 177.40) = 43.605, MSE = 16,259.051, *p* < .001, $\eta_{\rm partial}^{2}$ = 0.498. The main effects of *Group*, *F*(1, 44) = 0.008, MSE = 20,451.233, *p* = .930, $\eta_{\rm partial}^{2}$ = 0.001, and *Attacking Team*, *F*(1, 44) = 0.157, MSE = 9,806.853, *p* = .694, $\eta_{\rm partial}^{2}$ = 0.004, were not significant.

A significant two-way interaction of *Group* × *Attacking Team*, *F*(1, 44) = 14.879, MSE = 9806.853, *p* < .001, $\eta_{\rm partial}^{2}$ = 0.253, revealed that both groups responded faster when the attacking team wore the color of the preferred team. In particular, supporters of Borussia Dortmund responded faster when the yellow team attacked (*M* = 934 ms, SD = 139) than when the blue team attacked (*M* = 965 ms, SD = 144), and supporters of Schalke 04 responded faster when the blue team attacked (*M* = 933 ms, SD = 140) than when the yellow team attacked (*M* = 959 ms, SD = 158). The remaining interactions were not significant: *Group* × *Spatial Distance*, *F*(4.03, 177.40) = 1.066, MSE = 16,259.051, *p* = .375, $\eta_{\rm partial}^{2}$ = 0.024, *Attacking Team* × *Spatial Distance*, *F*(4.38, 192.66) = 1.319, MSE = 7,171.624, *p* = .262, $\eta_{\rm partial}^{2}$ = 0.029, *Group* × *Attacking Team* × *Spatial Distance*, *F*(4.38, 192.66) = 1.197, MSE = 7,171.624, *p* = .313, $\eta_{\rm partial}^{2}$ = 0.026.

### Discussion

Experiment 4 attempted to isolate a presumed “perceptual” variable, which might have overshadowed the effects of team preferences on offside judgments in Experiment 1. Therefore, as in Experiment 1, supporters of Borussia Dortmund and supporters of Schalke 04 judged offside in displays showing scenes of a (virtual) match between these teams. In contrast to Experiment 1, however, participants in Experiment 4 could not predict which team would attack in the next display because pictures showing the yellow team attacking and pictures showing the blue team attacking were randomly mixed within blocks. The main result of Experiment 4 was that the accuracy of offside judgments (at the smallest offside position of 1) was reduced for yellow as compared to blue forwards, and this effect was of similar size for supporters of both teams (cf. Fig. 9). These findings contrast with those of Experiment 1, where supporters of Borussia Dortmund, as compared to supporters of Schalke 04, had shown a bigger reduction in the accuracy of offside judgments for yellow compared to blue forwards.

The results of Experiment 4 provide further evidence for our account of the findings of Experiment 1, according to which a perceptual variable had—unexpectedly—overshadowed the effect of different team preferences on offside judgments. The perceptual variable should always decrease the accuracy of offside judgments for yellow as compared to blue forwards, whereas team preference should decrease the accuracy of offside judgments for the preferred team. We assumed that both variables are at work when the attacking team is predictable, as in Experiment 1, whereas only the perceptual variable is at work when the attacking team is not predictable, as in Experiment 4. The results of Experiment 4 confirmed these predictions.

## Footnotes

1 An interesting and illustrative collection of shirts from Borussia Dortmund can be found at https://bvb-trikotgeschichte.de/trikot-sammlung.

2 For pairwise comparison of two metrical variables, SPSS computes Student’s *t* test with uncorrected degrees of freedom (*df*) for the case of equal variances, and—apparently—the Welch test with corrected degrees of freedom for the case of unequal variances.

3 In the appendix, we report an additional experiment (Experiment 4), where we randomly varied the color of the attacking team instead of blocking the color of the attacking team as in Experiments 1 and 2.
